# Variations in vaccination uptake: COVID-19 vaccination rates in Swedish municipalities

**DOI:** 10.1371/journal.pgph.0001204

**Published:** 2022-10-20

**Authors:** Elis Carlberg Larsson, Emanuel Wittberg, Susanne Wallman Lundåsen

**Affiliations:** 1 Institute for Analytical Sociology, Linköping University, Norrköping, Sweden; 2 Centre for Local Government Studies, Linköping University, Norrköping, Sweden; Universidad Nacional de Colombia, COLOMBIA

## Abstract

Facing the threat of the ongoing COVID-19 pandemic, vaccines are important for limiting the spread and consequences of the pandemic. In this study, we provide a descriptive overview of the within-country variations of vaccine rates by examining to what extent voter turnout, support for an anti-establishment political party (Sweden Democrats), presence of first-generation immigrants, and Evangelical religiosity are associated with the within-country variation in vaccine uptake rates. We use official register data for municipality-level vaccine rates and municipality-level regressions with regional fixed effects. Our analyses show that vaccine uptake, on average, is lower in municipalities where the anti-establishment political party Sweden Democrats has higher vote shares and where a larger share of the population is first-generation immigrants. We discuss that potential explanations for these associations between vote shares for an anti-establishment party and shares of first-generation immigrants could be lower levels of trust in institutions and language barriers.

## Introduction

The COVID-19 pandemic is an ongoing and severe health threat at both the national and global levels. By the end of July 2022, the COVID-19 pandemic led to approximately 6.4 million deaths globally [1]. Similar to previous epidemics and pandemics [2, 3], COVID-19 has had considerable negative social and economic consequences [4–7]. Currently, vaccination is one of the safest and most efficient methods of preventing and reducing the spread of infectious diseases. As a result, vaccination has become one of the main tools against the severe consequences of the COVID-19 pandemic [8]. To keep death tolls as low as possible and minimize stress on the healthcare system, enough people need to be vaccinated, and successful rollout strategies need to be deployed [9]. For instance, estimations indicate that vaccine uptake would need to be between approximately 60% and 80% to achieve herd immunity against the original COVID-19 virus [10, 11]. The threat of the Omicron variant and possible future mutations makes it important to achieve as high a vaccination uptake as fast as possible to ease the pressure on the healthcare system, as well as to reduce the negative consequences of COVID-19 related to health and its social and economic effects.

Against this backdrop, local-level variations in vaccine uptake against COVID-19 can constitute a significant threat to public health, both at the national and global levels. Even when vaccines are free of charge and available, vaccination rates often tend to be lower among certain social groups, such as cultural, ethnic, supporters of radical right-wing political parties, and religious minorities [12–16]. Under certain circumstances, this may result in local clusters of low vaccine uptake. These local pockets with higher concentrations of unvaccinated individuals could enable a pandemic virus to survive [17, 18] and continue to mutate into variants. In other words, the persistence of local pockets can potentially threaten otherwise successful vaccination programs. Hence, it is important to investigate the factors that may contribute to the formation of local clusters of less vaccinated populations.

The present study aims to investigate the presence of within-country variation in vaccine rates across Swedish municipalities (the lowest administrative level in Sweden). In particular, the current study seeks to understand what characterizes local communities that have lower vaccine uptake. Toward this end, we study the within-country variation in vaccine rates by investigating the variation across Swedish municipalities (the lowest administrative level in Sweden). Sweden represents a tough empirical test because it can be considered a least likely case of noncompliance with vaccination from an international perspective, with the country having a long history of world-leading levels of voluntary compliance in vaccine programmes directed at children [19]. From a global perspective, it is also a wealthy country with a well-functioning healthcare system and a good supply of vaccines approved by the European Medicines Agency (EMA). In other words, if we find significant local-level variations within this context despite these favorable conditions, these patterns are likely to be found in other similar contexts as well. The variation in vaccine uptake across regions in countries with greater inequality in access to health care is likely greater and may also be driven by unequal access to health care in addition to refusal or hesitancy to vaccinate. Furthermore, by using analyses based on within-country variations, we avoid many of the confounders associated with cross-country comparisons because we, for instance, are dealing with variations within the same legislative and institutional healthcare context [20]. During the period included in the present study, neither mandatory vaccinations nor “vaccine passports” (for access to certain institutions or public spaces) were imposed (Later in the fall of 2021, Sweden implemented the usage of “vaccine passports” for larger events.). Instead, during the studied period, Sweden mainly relied on the population to voluntarily comply with recommendations to combat COVID-19. In this way, the Swedish case allows us to examine factors of vaccination uptake that are isolated from legislative factors that enforce vaccination.

We use high-quality Swedish administrative data at the municipality level to investigate which municipality characteristics are associated with different vaccine uptake rates. Our explanatory variables relate to factors that previously have been found to correlate with low trust in public institutions at both the individual and the community levels. They can therefore be seen as proxies for low trust in public institutions.

Our results reveal that the share of non-European immigrants and support for a Swedish anti-establishment (often classified as radical right) party, the Sweden Democrats (SD), in a municipality are both negatively associated with vaccine uptake at the municipality level. These findings indicate that the negative consequences of pandemics such as COVID-19 may be larger in these local communities. Lower vaccine uptake in municipalities with larger shares of immigrants could potentially enhance the negative consequences of COVID-19 for different immigrant groups, as research has shown that certain immigrant communities have had an increased risk of hospitalization and mortality during the COVID-19 pandemic [21, 22]. In the same sense, lower vaccine uptake in municipalities where the support for SD is strong would likely increase the negative consequences of COVID-19 for the residents of these municipalities. Concurrently, even if we use municipality-level data, a lower vaccine uptake among groups voting for the Sweden Democrats is not implausible and would follow an international trend where supporters of anti-establishment parties tend to have a lower institutional trust while also having lower trust in science [23] and being more reluctant to vaccinate [14, 24]. Recent data have shown that the relative risk of needing intensive care treatment following a COVID-19 infection differed across geographical contexts in Sweden. Residents in poorer rural towns and residents in poorer and more diverse metropolitan areas had a higher risk of needing treatment within an intensive care unit if infected by COVID-19 [25].

Measures taken to fight a pandemic depend on a functioning national public health infrastructure capable of identifying, tracing, managing, and treating cases while also coordinating efforts efficiently [2]. Variations in vaccine uptake within a country with high access to vaccines that are free of charge are to a larger extent driven by the degree of compliance with recommendations to vaccinate, compared with variations in vaccine uptake in countries with large inequalities in access to health care and vaccines. As the COVID-19 pandemic and previous pandemics have shown, many countries are inadequately prepared to mitigate and suppress ongoing pandemics [26, 27]. Thus, all countries must learn from the experience of the COVID-19 pandemic to prepare for new—and possibly even worse—epidemics or pandemics in the future [28, 29].

The paper’s contribution is threefold. First, our results add new evidence to the notion that local pockets of groups that are likely to have lower levels of institutional trust and language barriers can constitute important threats to the fight against both COVID-19 and future pandemics. Second, our paper shows that considerable variations in vaccination uptake can emerge at the subnational level, even in a wealthy country with relatively limited subnational variation in access to the health care system. Third, our results from the context of Sweden–a country with a high quality of government and a long history of successful vaccination programs–suggest that vaccine uptake rates are not solely dependent on the quality of public institutions. Therefore, it is urgent to find new policy measures that reduce the gap in vaccine uptake between different local communities. The paper proceeds with a theoretical background and formulation of our hypotheses, a brief description of the Swedish case, and a data and methods section. We then present the results and conclude with a discussion of the results.

## Theoretical explanations for why vaccine uptakes vary within a country

As we focus on variations within a single country, we are operating in a context with the same set of legal institutions and, in the Swedish case, similar levels of access to health care within the country. To understand why the vaccine uptake rates vary within a country, we also need to understand its flip side, that is, the concept of vaccine hesitancy or refusal. Formally, *vaccine hesitancy* can be defined as the “delay in acceptance or refusal of safe vaccines despite availability of vaccination services” [30]. We recognize that the decision to vaccinate may be a gradual process for each individual; however, an individual’s decision not to vaccinate (or to delay their vaccination) will contribute to the aggregate local-level vaccine rates. The technology used for some of the vaccines used during the COVID-19 pandemic was novel, which may have contributed to an initial skepticism toward their safety [31]. This could potentially make vaccine hesitance more pronounced for the COVID-19 vaccine compared with other vaccines. However, these concerns about the safety of the vaccines are more likely to persist within certain groups [31]. Previous studies that have used individual-level data indicate that certain groups are either more reluctant in their *intent* to vaccinate or have lower levels of actual vaccine uptake against COVID-19 [32–35]. The existing local differences in vaccine uptake may also be due to differences in local social norms regarding hesitancy or refusal to vaccinate. If groups that on average are more likely to have lower levels of vaccine uptake live close to one another, this may enhance the difference between local communities [36, 37]. Therefore, we present an overview of previous studies that have identified important factors for vaccine hesitancy.

In a systematic review, Truong and colleagues [31] identify seven major factors that affect vaccine hesitancy and acceptance: demographic factors, accessibility and cost, personal responsibility and risk perceptions, precautionary measures taken based on the decision to vaccinate, trust in health authorities and vaccines, the safety and efficacy of a new vaccine, and a lack of information or vaccine misinformation. Jennings and colleagues [34] further elaborate on the individual mechanisms behind the reluctance to vaccinate and suggest three main (internal) drivers of vaccine hesitancy: (1) beliefs in conspiracy theories, (2) concerns about side effects, and (3) religious beliefs. Furthermore, studies have shown that socioeconomic factors such as levels of education may explain the willingness to vaccinate [37]. Individuals may also be influenced by exposure to COVID-19 from media consumption, which may affect their perceived susceptibility to the disease [38].

The belief in conspiracy theories refers to skepticism about the commercial and societal motives of both the vaccine and the virus itself, which may result in a lower willingness to vaccinate [33, 39, 40]. Another set of explanations hinges on concerns about the negative side effects of the vaccine. Individuals with these concerns often stress that the vaccines were rushed and, therefore, that the vaccines are untested and unsafe because of potentially unknown negative (long-term) side effects [11, 12, 41]. Concerns about side effects may be grounded in actual side effects, but the likelihood and severity of these side effects are often exaggerated, especially compared with the negative effects and likelihood of becoming seriously ill from the disease itself [42].

Additionally, individuals with certain religious beliefs, that is, religious beliefs that are more dogmatic in nature (i.e., religious rules ought to serve as strict guidelines for daily life), have in general been shown to be more reluctant to vaccinate [43]. This is argued to arise both from religious beliefs about diseases being a natural part of life and from preferring to rely on spiritual alternatives to vaccines [44].

It is important to note that even if we have listed the main explanations of vaccine hesitancy (or refusal) identified in the literature, they are not mutually exclusive; rather, they tend to overlap. The main drivers of vaccine hesitancy within a population may also be unevenly distributed geographically due to, for instance, residential segregation. Residential segregation can in turn contribute to the formation and enhancement of local clusters of less vaccinated inhabitants.

### Differences in vaccine uptake across social groups

In this section, we formulate hypotheses regarding which social groups can be expected to have a lower vaccine uptake based on previous research.

The literature indicates that a common denominator among those who are skeptical about vaccines is low trust in government institutions and public health agencies [37, 45, 46]. Those with low levels of trust in government institutions are also on average less willing to comply with COVID-19 recommendations and restrictions [47]. Distrust in government institutions and public health agencies also increases the likelihood of becoming a target of disinformation and facilitates beliefs in conspiracy theories [33, 48]. Moreover, if groups with low trust in government institutions tend to live or work in proximity to each other, these attitudes could become accepted and considered the local “norm” [36]. Hence, vaccine hesitancy might spread and be reinforced, especially within segregated social networks.

The literature points to trust in government institutions as a key underlying explanatory variable for compliance with government recommendations to vaccinate [11, 34, 49]. In the present study, we cannot rely on survey measurements of trust in government institutions because no survey produces reliable measurements for all 290 Swedish municipalities. However, trust in government institutions tends to correlate positively with voter turnout [50]. Therefore, voter turnout could serve as a proxy for trust in government institutions at the local level.

Based on this, our first hypothesis on the relationship between trust in government institutions—as proxied by the voting turnout—and vaccine uptake can be formulated as follows:

***Hypothesis 1A***: *Voter turnout is expected to correlate negatively with vaccine uptake at the municipality level*.

Another group that often stands out as having low trust in both government institutions and science is supporters of populist parties [14, 23, 24, 33]. These voters often have considerably lower trust in government institutions compared with the population average [51, 52]. This is also reflected in the rhetoric among populist or anti-establishment parties, which frames these parties as if the “common people” are being deceived by an “elite” [53]. The elite are argued to favor their interests, which are not aligned with the interests of the common people [14, 52–54]. Hence, the lower level of trust in government institutions is argued to be a result of the distrust, built-in antagonism, and anti-establishment sentiment of these political parties and that they are more likely to attract groups of voters with lower levels of trust in public institutions [14, 52, 55].

We argue that the share of anti-establishment voters could serve as an additional proxy for trust in government institutions. In Sweden, the Sweden Democrats (SD) stand out as the largest anti-establishment political party [55–59], and their voters are considerably less trusting of government institutions compared with the voters of other parties [51]. Survey data indicate that although the levels of trust in government institutions became less polarized and even increased during the initial phase of the pandemic [60], political trust remained considerably lower among voters of SD [51]. Therefore, it is likely to assume that the presence of SD voters could serve as a proxy for trust in government institutions. This is also supported by survey results indicating that SD supporters more often believe in conspiracy theories about COVID-19 (and in general) and are more skeptical of the vaccine [61]. Moreover, survey data from January 2021 showed that the supporters of SD expressed a markedly lower willingness to vaccinate [62]. Hence, our next hypothesis can be formulated as follows:

***Hypothesis 1B***: *The vote share of the SD is expected to negatively correlate with vaccine uptake at the municipality level*.

Previous studies have also shown that ethnic minorities, especially immigrants from Asia and Africa, often have lower vaccine uptake and higher vaccine hesitancy than the general population in Western countries [12, 63, 64]. Figures from the register on vaccination uptakes of the Public Health Agency of Sweden also show that non-European immigrants tended to have lower levels of vaccine uptake, especially during the first phases of the COVID-19 vaccination program [35]. Language barriers have been suggested to hinder access to information about vaccines from government institutions, hence explaining the lower levels of vaccine uptake [65]. Additionally, levels of social and institutional trust are on average lower among immigrant groups [66]. Additionally, immigrant dense communities in Sweden are more often socioeconomically relatively disadvantaged [67]. Furthermore, previous studies have also found that immigrants—partly because of their often more vulnerable position in societies—trust government institutions less, and this also includes less trust in healthcare providers [11, 32, 68, 69]. The presence of ethnic minorities could be a proxy for groups that are harder to reach because of language barriers and distrust in government institutions.

Based on this, we can formulate the following hypotheses:

***Hypothesis 2A*:** *The share of the population with a first-generation immigrant background is expected to correlate negatively with vaccine uptake at the municipality level*.
***Hypothesis 2B*:** *The share of non-European immigrants is expected to correlate negatively with vaccine uptake at the municipality level*.

As mentioned above, religious beliefs may also be associated with vaccine skepticism and a higher tendency to believe in conspiracy theories [43, 70]. This could be connected to a general reluctance among certain religious groups toward modern medicine, where religious teachings have prioritized prayers over COVID-19 treatments and vaccines [44]. Hence, some religious groups are more inclined to rely on alternative approaches (e.g., holy water and prayers) as a way of combating contagious diseases. There is also empirical evidence of lower vaccine willingness among certain Christian communities, such as Evangelical Christians [71]. Even though religious diversity is increasing in Sweden, mainly because of immigration, Christianity is still predominant in the population [72]. A more fundamentalist view of religion, which is more likely to be found among, for instance, Evangelical Christians, also tends to be associated with less prosocial values [73]. We acknowledge that religious affiliation is just one component of religious belief; however, we can assume that a higher presence of Evangelical Christian religiosity in a municipality will be associated with lower vaccine uptake. Hence, we formulate the following hypothesis:

***Hypothesis 3*:** *The share of the population affiliated with Evangelical churches is expected to correlate negatively with vaccine uptake at the municipality level*.

Together, these five hypotheses test to what extent within-country variations in vaccine rates are associated with trust in government institutions, the local presence of groups that are harder to reach, and Evangelical religiosity.

### The case of Sweden

Our empirical focus is on the case of Sweden, which is a relatively small (population of approximately 10 million) and wealthy country with comparatively high levels of trust in government institutions [50]. Sweden was subject to much international attention during the COVID-19 pandemic. It became an example of a global outlier, particularly during the first phase of the pandemic during the spring of 2020, using measures to fight the pandemic mostly based on voluntary cooperation rather than strict lockdowns. Studies have found a rallying effect even in Sweden, where trust in government institutions increased during the spring of 2020 [60].

A similar voluntary approach has been used in the Swedish vaccine program, where these are based on voluntary compliance. The voluntary compliance approach to vaccinations is based on previous experiences with world-leading levels of vaccine uptake among children [19]. The vaccines have been free of charge, and public authorities have encouraged all individuals aged 16 and above to vaccinate (The recommendation to become vaccinated was during the fall 2021 reduced several times to the age of 12. However, for the time period that is being examined in this study, the recommendation to become vaccinated was for all individuals aged 16 or older.). Many municipalities and healthcare providers have, in cooperation with civil society organizations, attempted to raise awareness of the importance of vaccinating through public information campaigns and targeted campaigns in different languages to immigrant communities. In addition, different groups were vaccinated according to a predefined order determined based on age and health condition. Until September 2021, the vaccines deployed in Sweden were Comirnaty by Pfizer–BioNTech, Spikevax by Moderna, and Vaxzevria by Oxford–AstraZeneca. These vaccines have been approved by the EMA [74–76]. In September 2021, all individuals 16 years or older were offered to vaccinate against COVID-19. The vaccination program has been managed at the county level, following how the healthcare system is organized in Sweden. This resulted in some variations in how the campaigns to get people vaccinated were organized. We describe in the Data and Method section how we addressed the regional variation in our models through county fixed effects.

## Data and method

Our data are aggregated for all 290 Swedish municipalities during 2019, except for election data and vaccination data, which are from 2018 and 2021, respectively. Our data do not contain any individual-level information and, therefore, are less sensitive from an ethical perspective. Moreover, the data on vaccination against COVID-19 were both anonymized and aggregated by The Public Health Agency of Sweden before we accessed it.

Municipalities represent a small but aggregated geographic unit for which we can observe variations in vaccine uptake. The median size of Swedish municipalities is approximately 15,978 inhabitants, which may be small enough to identify local variations (We acknowledge that there may be considerable variation in vaccine uptake within municipalities, especially the most populous metropolitan areas (Gothenburg, Malmö, and Stockholm). Further studies of the variation within these metropolitan areas are needed.). Municipalities are also relevant because a large share of the public institutions and execution of welfare state services are tied to municipalities, such as elderly care, childcare, and schools. Therefore, municipalities will often have to intervene and adopt policies if there are large outbreaks of COVID-19.

### Dependent variable

To capture *vaccination uptake*, we use the population share in each of Sweden’s 290 municipalities that were vaccinated with two doses by September 16, 2021, as reported by the Swedish Public Health Agency [77]. This is an appropriate time point for data collection because the entire adult Swedish population had been offered two doses of vaccine at that point, and it was shortly before a third vaccine dose was offered to the elderly and high-risk groups in Sweden. The shares who have been fully vaccinated are estimated using the population consisting of all inhabitants who resided in Sweden and were 16 years old on December 31, 2020. This demographic information is obtained from Statistics Sweden.

To account for potential age effects arising from the fact that old age is a risk factor for COVID-19, which can have caused the elderly population to be prioritized in vaccination programs, as well as having more motives to be vaccinated due to higher risks of severe illness, we age-standardized the dependent variable (We age-standardize the vaccination using nine age groups (16–17, 18–29, 30–39, 40–49, 50–59, 60–69, 70–79, 80–89, and 90+)). This allows us to analyse municipalities without the results being distorted by different age structures across municipalities or by the effect of age itself.

**Fig 1:**
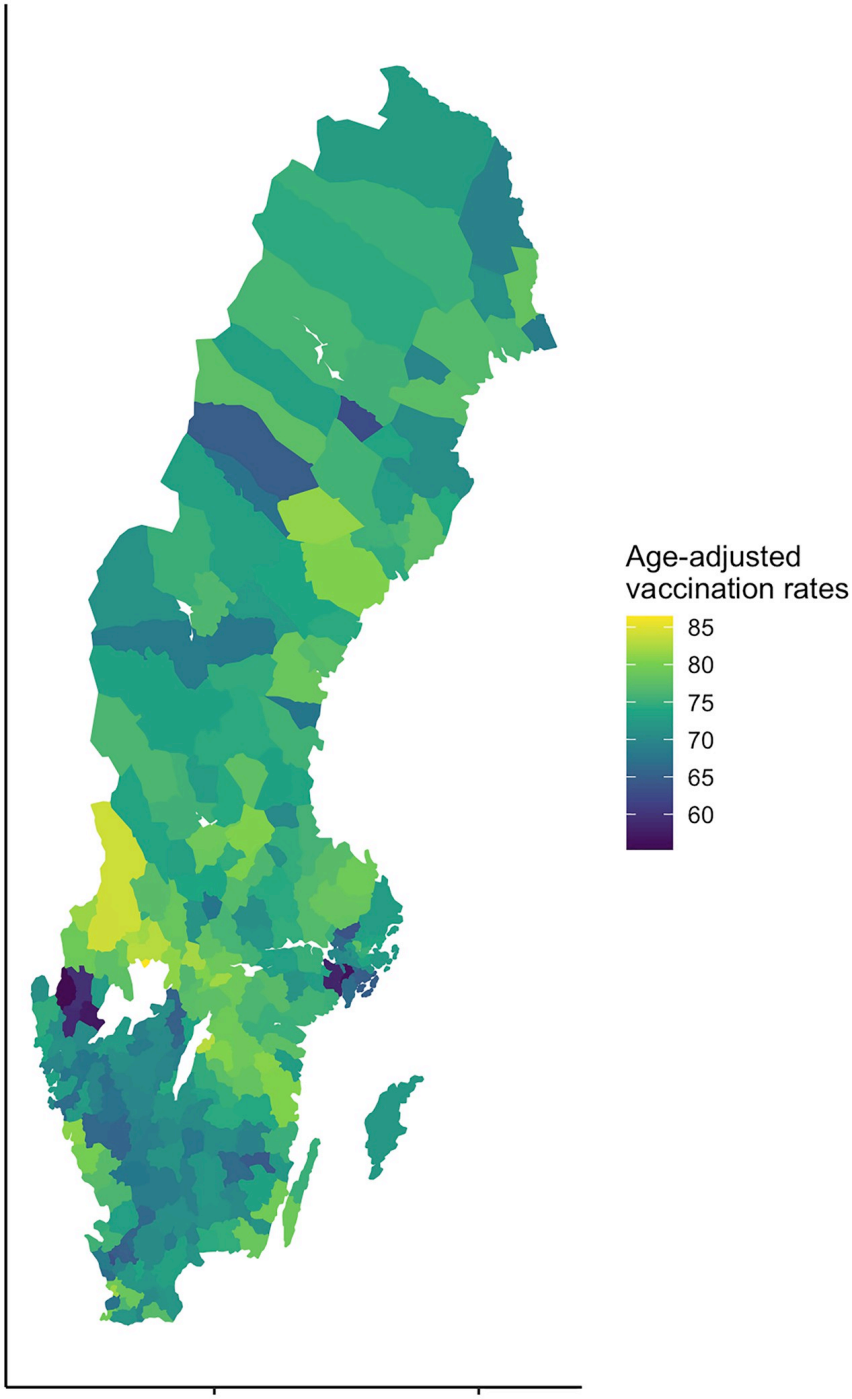
Map of Sweden where municipalities are colored based on age-adjusted vaccination rates (in %). Source: Statistics Sweden and the Swedish Public Health Agency. Map layer: SWEREF99 (https://www.lantmateriet.se/oppnadata).

Descriptive analyses highlight that there is substantive variation across municipalities in vaccine uptake. In Fig 1, the age-adjusted vaccination uptake for each municipality in Sweden is visualized on a map. The figure shows that even if we lose some granularity using aggregated municipality data, there are substantive intermunicipal variations that can be studied using our chosen design.

### Independent variables

We use several independent variables to test our formulated hypotheses. We use two different variables to serve as proxies for trust in government institutions: voter turnout and share of votes for the SD. *Voter turnout* is the share of eligible voters who voted in the 2018 Swedish election to the national parliament, “Riksdagen,” within each municipality. *SD vote share* is the share of votes the SD received in each municipality in the 2018 election to Riksdagen. Both variables are from the Swedish Election Authority’s (2021) official reports. From Statistics Sweden [78], we collect information about the *share of immigrants* (i.e., born outside Sweden) in each municipality. The data are categorized so that we can tap both the total share of immigrants in each municipality and describe the immigrant group according to regions of birth, namely, *European immigrants* and *non-European immigrants*.

Similar to political trust, there are no available survey data that cover religiosity (belief) in all Swedish municipalities. Therefore, we must rely on proxy variables to capture *religiosity* within each municipality. We use data collected for the Free Church Report (in Swedish: *Frikyrkoundersökningen*) [79], which reports the total share of members in Evangelical (or leaning toward an Evangelical interpretation of Protestantism) in each Swedish municipality. We use free church membership rates because we believe that so-called free Protestant churches (i.e., not the former Swedish state church) could serve as a better proxy for the more intense religious beliefs that could be conducive to vaccine hesitancy. Admittedly, this is not a perfect measurement of Evangelical Christianity because the group of “Free Churches” also contains more mainstream Protestant congregations, but it is used as a first empirical test of the role of religiosity at the municipal level.

In the S1 Fig, the bivariate associations between all our independent variables and vaccine uptake are visualized.

### Control variables

We also include several control variables in our models that capture socioeconomic and demographic differences across municipalities. From the Swedish Association of Local Authorities and Region’s database [80], we use the *unemployment rates* for each municipality. To capture *income* differences, we use the logged median annual income for each municipality, and to capture *low levels of education*, we include the shares of the population in each municipality who did not finish postsecondary education (the Swedish gymnasium) (We also tried to estimate models with other specifications of the education variables, such as a dummy for the share of citizens that have a completed university education or to add a variable for the share of inhabitants with high school education, in addition to our current variable. Our main result is consistent for all specifications.). Finally, the *logged population size* of each municipality is included to account for scaling effects across municipalities [78].

S1 Table, found in the supporting information, includes descriptive statistics of all the variables included in the analyses.

### Analytical approach

To analyze the patterns of vaccine uptake at the municipal level, our main model is an OLS regression model with fixed effects at the county level. The decision to use OLS as our main model deserves motivation given that our dependent variable is on a percentage scale while OLS assumes a continuous dependent variable. While we acknowledge the fact that OLS should be used with caution on percentage data, OLS nevertheless serves as a practical choice in many situations. OLS often performs well when used on percentage data as long as the sample size is large and the predicted values do not approach the edges of the scale (0 or 100) [81]. Using a dependent variable in percentage format is foremost a problem when the observed values approach the edges of 0 or 100. In these cases, there is a risk that an OLS estimation results in predicted probabilities below 0 or above 100. These problems are limited in our specific case. As shown by our descriptive table in the S1 Table, 75 percent of all Swedish municipalities had an observed vaccination rate (2 doses) of 76 or lower. The highest observed vaccination rate was 86, and the lowest was 55. Moreover, none of the vaccine uptakes predicted by our OLS models are close to the edge of the percentage scale, and the estimated distribution closely resembles the observed distribution of the dependent variable (S5 Fig). This indicates that our dependent variable approximately behaves like a continuous variable within the range of observed values between 55 and 86. Despite this, we also estimated beta regression [82] as a robustness test, and these results are included as S7 Table and discussed under the subsection *Robustness checks* of the *result* section. Beta regression restricts the dependent variable to interval 0–1 and is, therefore, one of the most reliable models for estimations of models with a dependent variable on a percentage scale. In short, the results obtained using beta regression are substantially the same as the results from OLS. In addition, as shown in S5 Fig, the distribution of predicted vaccine uptakes is almost identical for the OLS estimation and the beta regressions. Given the similar results of OLS and beta regression, we have, for simplicity reasons, chosen to use OLS as our main model in the results section.

Our estimations include county-fixed effects for two reasons. First, county-fixed effects can account for some spatial dependencies in vaccine hesitancy. It is also likely that spatial effects influence the included independent variables, such as SD voter share or voter turnout. Second, the healthcare system in Sweden, including COVID-19 vaccinations, is managed and administered by the counties. Differences in willingness to vaccinate across counties, as well as opportunities to vaccinate, are likely partly a result of regional differences in healthcare. In addition, the administration of the vaccination program at the county level has resulted in a variety of policies and operationalizations of the vaccination program carried out across regions. The purpose of our paper is to identify factors that are associated with vaccination variation within regions; therefore, it goes beyond the limited scope of the present study to attempt to also identify correlates of regional differences. However, to produce unbiased estimates, we still account for policy differences at the regional level by including fixed effects at the county level.

Hence, our models can be described using the following equation:

$$Y_{i}=\beta_{0}+\beta_{1}X_{i}+\sum_{j=2}^{j=n}B_{j}C_{i}+d_{C}+\varepsilon_{it}$$

Where Y_*it*_ is the dependent variable, vaccine uptake, and X_i_ denotes our independent variable(s). *i* indexes municipalities, β_0_ is the global intercept, and d_*C*_ are the county-specific dummies. C_i_ denotes municipal-level control variables, and B_j_ denotes their coefficients. Ε_i_ is the error term.

Prior to running the regression analyses, we also explored the data and investigated the variable distributions and associations. We visualized scatterplot matrices for all combinations of variables to search for potential nonlinearities and outliers in the data. No nonlinear patterns were observed (S1 Fig), which motivated us to model a linear relationship between the dependent variable and our independent variables. We also investigated the correlations among the included variables (S2 Fig and S2 Table). Some variables are moderately to highly correlated with each other, which is common for analyses of aggregate-level data concerning municipalities and regions. To avoid our results being influenced by multicollinearity, we also performed variance inflation factor (VIF) tests for all models. All VIF values were below the commonly accepted thresholds, indicating that multicollinearity should not have influenced the results (in the main analyses, we include outliers because these may provide unique and relevant information about the studied relationship. However, to ensure that our results are not driven by extreme values, we have also performed analyses where outliers are excluded. These analyses show that the main patterns presented in the current paper are robust to outliers. The only discrepancy observed is that election turnout becomes statistically significant, a variable that shows inconsistent results across other robustness controls (see S3 Table). Additionally, as shown by residual plots found in S3 and S4 Figs, the residuals from our main model do not suffer from heteroskedasticity or other forms of systematic bias.

In the main analyses, we include outliers because these may provide unique and relevant information about the studied relationship. However, to ensure that our results are not driven by extreme values, we have also performed analyses where outliers are excluded. These analyses show that the main patterns presented in the current paper are robust to outliers. The only discrepancy observed is that election turnout becomes statistically significant, a variable that shows inconsistent results across other robustness controls (see S5 Table).

## Results

We performed OLS regression models with county-level fixed effects. This allowed us to test our formulated hypotheses. Table 1 presents the regression models.

**Table 1:** The association between various independent variables and vaccine uptake estimated with OLS regression.

	Model 1	Model 2	Model 3	Model 4	Model 5	Model 6	Model 7	Model 8
SD voter share	-0.135*				-0.182**	-0.202**	-0.138*	-0.207**
	(0.064)				(0.063)	(0.064)	(0.063)	(0.064)
Election turnout		0.545***			0.235	0.394***	0.449**	0.277
		(0.106)			(0.145)	(0.113)	(0.141)	(0.146)
Members in free church			0.007		-0.076	-0.056	-0.095	-0.058
			(0.097)		(0.093)	(0.093)	(0.094)	(0.093)
Share foreign-born				-0.222***	-0.179**			
				(0.041)	(0.057)			
Share born outside Europe						-0.305***		-0.309***
						(0.088)		(0.088)
Share born in Europe							-0.081	-0.091
							(0.074)	(0.073)
**Control variables**								
Unemployment rate	-0.215	-0.153	-0.208	0.068	0.021	0.198	-0.181	0.191
	(0.130)	(0.126)	(0.132)	(0.135)	(0.138)	(0.162)	(0.126)	(0.162)
Log(median income)	7.884*	-0.457	7.551*	5.396	2.698	2.505	0.281	3.243
	(3.229)	(3.466)	(3.256)	(3.115)	(3.548)	(3.494)	(3.513)	(3.540)
Log(population size)	-0.633*	-0.053	-0.472	0.079	-0.054	0.093	-0.210	0.083
	(0.254)	(0.247)	(0.245)	(0.254)	(0.257)	(0.265)	(0.257)	(0.265)
Share with low education	-0.796***	-0.755***	-0.954***	-0.816***	-0.538***	-0.494***	-0.591***	-.495***
	(0.143)	(0.123)	(0.123)	(0.120)	(0.142)	(0.143)	(0.143)	(0.143)
Constant	-11.341	37.856	-9.223	14.580	28.785	14.465	39.989	16.486
	(41.552)	(40.951)	(41.935)	(40.035)	(40.289)	(40.579)	(40.877)	(40.566)
Observations	290	290	290	290	290	290	290	290
R^2^	0.721	0.742	0.716	0.744	0.756	0.758	0.748	0.760

***Notes*:** Unstandardized coefficients; robust standard errors within parentheses.

Significance:

*p < 0.05;

**p < 0.01;

***p < 0.001.

All models include county fixed effects.

### Turnout, support for anti-establishment parties, and vaccine uptake

The first two hypotheses refer to the relationship between vaccine uptake and our proxies of trust in government institutions. We hypothesized that (H1A) *municipalities where voter turnout is lower will*, *on average*, *have a lower vaccine uptake* and that (H1B) *municipalities where the SD have a higher vote share will*, *on average*, *have a lower vaccine uptake*.

In Table 1, Model 2, a positive association between voter turnout and vaccine uptake is identified (*p* < 0.001). However, the association is not statistically significant when the other independent variables are introduced (Table 1, Model 5). In other words, H1A cannot be fully corroborated.

**Fig 2:**
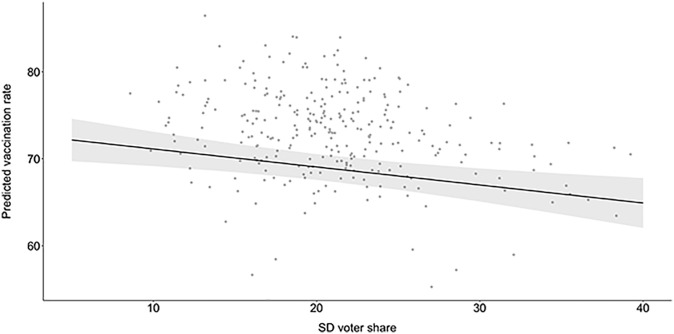
Predicted vaccination rates for municipalities with various levels of support for the SD. Gray dots are observed values.

Next, we focus on H1B, which posits that on average, vaccine uptake will be lower in municipalities where the SD have a higher vote share. As shown by the models in Table 1, a statistically significant negative association between the vote share of SD and vaccine uptake, in line with H1B, is observed: an increase of 1% in SD voter shares in a municipality, on average, decreases vaccine uptake by 0.135% (*p* < 0.1). This association is robust over different specifications and the estimated coefficient increases when other interdependent variables are introduced in Model 5 β = -0.182, (p<0.01). When we plot the marginal effects of this correlation in Fig 2, it becomes clear that this marginal effect is meaningful. An increase in SD’s vote share of 10 percentage points is estimated to be associated with almost 2 percentage points lower vaccine uptake in the municipality. In sum, these results provide support for H1B, indicating that vaccine uptake in Swedish municipalities correlates negatively with the share of anti-establishment voters.

### Share of immigrants and vaccine uptake

We formulated two hypotheses regarding the share of first-generation immigrants residing in a municipality and its association with vaccine uptake in the municipality. First, we hypothesized that (H2A) the *share of the population being first-generation immigrants would correlate negatively with vaccine uptake*. Second, we formulated a more granular hypothesis regarding the region in which a migrant was born. More precisely, we hypothesized that (H2B) *the share of non-European immigrants would correlate negatively with vaccine uptake at the municipality level*. In other words, we argue that the predicted association between the share of first-generation immigrants and vaccine uptake is expected to be mostly driven by the share of individuals born outside of Europe, mainly because of language cultural, and cultural barriers.

In Models 4 and 5, as shown in Table 1, we find support for H2A, that is, a negative association between the share of immigrants and vaccine uptake in Swedish municipalities. The association is statistically significant in both the bivariate (Model 4) and multivariate (Model 5) setups. In Model 5, which includes all control variables, a 1% increase in a municipalities’ share of immigrants, on average, decreases vaccine uptake by 0.179% (p < 0.01). As illustrated in Fig 2A, a 10 percentage point increase in the share of first-generation immigrants is estimated to be associated with 1.79 percentage point lower vaccine uptake at the municipality level. This provides support for our formulated hypotheses that municipalities, where a higher share of the population is first-generation immigrants, will, on average, have lower vaccine uptake.

However, when performing analyses on more granular groups of foreign-born individuals, we only find statistically significant associations for immigrants born outside Europe. In Table 1, Model 8, we can observe that the share of first-generation immigrants from a non-European country is negatively associated with vaccine uptake in municipalities. More precisely, an increase of 1% of foreign-born individuals in the municipality, on average, decreases vaccine uptake by 0.305% (p < 0.001). Meanwhile, we find no statistically significant association for the second migrant group: the share of individuals born in a European country. Moreover, the inclusion of these more granular immigrant groups does not have a substantive impact on the estimated coefficients of other independent variables. As expected, some fluctuations in the estimated coefficients are observed; all in all, they do not change the previously reported findings.

**Fig 3:**
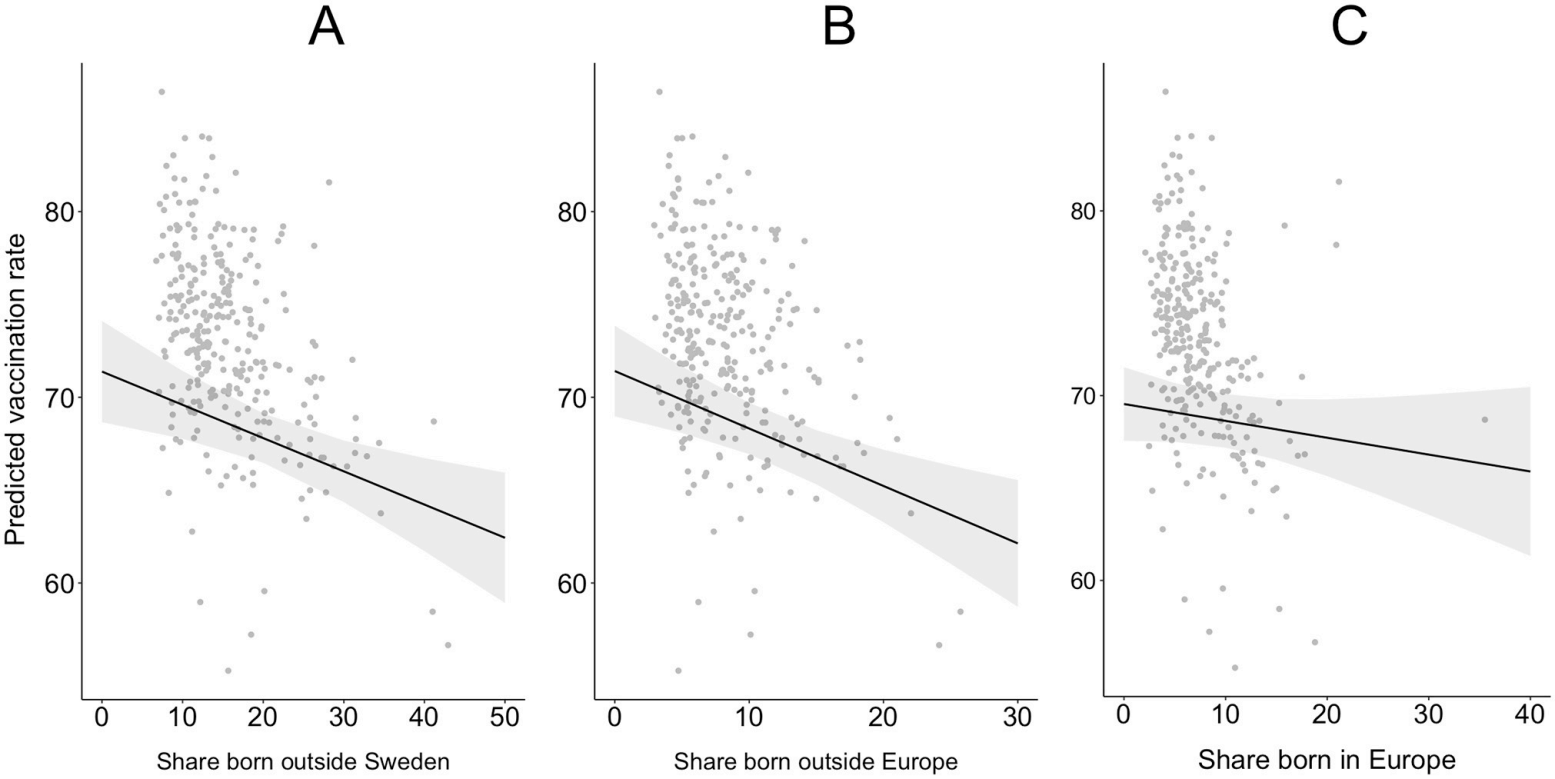
Predicted vaccination rates for municipalities with varying degrees of inhabitants born outside Sweden. **A)** Predicted vaccination rates and the proportion of all immigrants born outside Sweden. **B)** Predicted vaccination rates and the proportion of all immigrants born outside Europe. **C)** Predicted vaccination rates and the proportion of all immigrants born in Europe. Gray dots are observed values.

In sum, we find support for Hypothesis 2A. Municipalities with a higher share of non-European immigrants will, on average, have a lower vaccine uptake. The marginal effects of all of these variables are visualized in Fig 3A–3C, which illustrates how the correlation between population shares according to regions of birth and vaccine uptake varies depending on the granularity of the region of birth variable.

### Religiosity and vaccine uptake

We hypothesized that *the share of the population affiliated with Evangelical churches would have a negative correlation with vaccine uptake at the municipality level*. Neither our bivariate analyses nor the multivariate analyses provide any evidence of any such association. In other words, we observe no statistically significant associations between church membership and vaccination rates in a municipality. This means that H3 is not supported. The share of residents affiliated with Evangelical congregations is not associated with vaccine uptake at the local level in Sweden.

### Robustness checks

We also performed three types of robustness checks. In the first robustness check, we controlled for the different degrees of compliance with recommendations to vaccinate. Hence, we performed equivalent analyses using the age-standardized vaccination rate for one administered dose of vaccine (imperfect compliance) instead of two doses of the vaccine (S5 Table). The results are in line with those presented in Table 1. The only difference when using the share of the population that has taken one dose of vaccine is that voter turnout is statistically significant, where an increased voter turnout is associated with a higher one dose of vaccine uptake. No other substantial discrepancies can be observed.

Second, we also conducted a second robustness test in which Models 1–8 were estimated without control variables that are likely suspects of being fully or partly intermediate variables to some of our independent variables. The control variables such as *unemployment rate*, *median income*, and *share with low education* may be intermediate variables, and their inclusion may risk biasing the estimates downwards somewhat. On the other hand, these variables may also be confounders rather than only intermediate variables, and with the currently available data, we cannot distinguish these associations clearly. For instance, being an immigrant may increase the risk of unemployment, which, in turn, may affect the probability of committing to official vaccine recommendations. If this were the case, the estimated coefficient for the variable share with foreign backgrounds would be biased downwards. It is, therefore, relevant to conduct a sensitivity analysis to examine how the coefficient of the independent variables is affected when potentially intermediate variables are excluded. Estimations of Models 1–8 without potentially intermediating variables are presented in the S6 Table. The results show patterns similar to those presented in Table 1 but with larger regression coefficients. The only substantial difference is that a municipality’s vaccination rate has a statistically significant association with voter turnout.

Last, we performed beta regressions [82] on Models 1–8 to ensure that our result is robust even when we estimate models that have a link function that restricts the dependent variable to the interval 0–1 (or 0–100 percent). A limitation of OLS is that it assumes that the dependent variable is on a continuous scale. This may sometimes be a problem for dependent variables expressed as a percentage given that such a variable has an upper and lower limit. However, OLS may still be an acceptable approximation in cases when both the observed and the predicted values do not approach the edges of the percentage scale (0 or 100). Assuredly, none of the predicted vaccination uptakes from the OLS models was close to the edge of the scale, which indicates that OLS is a reasonable approximation for our study (see S5 Fig). Nonetheless, it is important to ensure that our results hold water in alternative model specifications. We, therefore, replicated our main models with beta regressions, and these results are presented as S6 Table. As expected, the results from the beta regressions are substantially similar to the main results obtained from OLS with regard to both direction, magnitude, and significance. Moreover, as shown in S5 Fig, the overall distribution of predicted vaccination rates for the beta regressions is very similar to the distribution of predicted vaccination rates for the OLS results. Thus, we can conclude that our main results are robust to several different model specifications.

## Discussion and conclusions

The current study set out to identify factors associated with the within-country variation in vaccine uptake related to COVID-19. To investigate this, we used age-standardized data on vaccine uptake rates among all 290 Swedish municipalities. This can be considered a tough empirical test because the general willingness to participate in vaccine programs has previously been very high in Sweden [83, 84]. We formulated five different hypotheses based on the role of trust in government institutions, the presence of hard-to-reach groups (i.e., immigrant groups), and religiosity. Our results add new evidence to the notion that local pockets of groups that are more likely to have low political and institutional trust may be an alarming obstacle to the fight against both COVID-19 and future pandemics.

First, our analyses highlight that in line with our hypothesis, the variation in local-level vaccine uptake was negatively associated with the share of anti-establishment voters. On average, vaccine uptake was lower in municipalities where the anti-establishment political party SD had a higher voter share. In our study, electoral support for SD served as a proxy for low levels of trust in government institutions. The SD has not explicitly used political rhetoric against COVID-19 vaccines, unlike many other populists and anti-establishment political parties in Europe; however, our data showed a negative association between their vote share and vaccine uptake at the municipality level. Our findings are in line with data from other countries that have found an association between local-level support for populist/anti-establishment political parties and the local-level vaccine uptake [14, 24]. Our interpretation of the association between the share of anti-establishment voters and vaccine uptake is also supported by other findings from individual-level survey data showing that Swedish anti-establishment voters are less willing to vaccinate [62].

Second, our analyses indicate that the share of first-generation immigrants, especially the share of first-generation immigrants born outside of Europe, is negatively associated with vaccine uptake at the municipality level. The lower levels of vaccine uptake in more immigrant-dense local communities may potentially have severe consequences given that previous studies have found increased risks of death, recovery in intensive care, and hospitalization from COVID-19 among the non-European immigrant population in Sweden [21]. Lower vaccine uptake among already vulnerable groups risks a further increase in preexisting (health) inequalities between immigrant groups and the native population. Overall, our paper shows that considerable variations in vaccination uptake can emerge at the subnational level even in contexts with only limited within-country variation in the quality of institutions. This negative association is likely driven by language barriers to accessing the information on vaccination programs and by other factors, such as lower levels of trust in public institutions [32].

The association between our other proxy for trust in government institutions—that is, voter turnout and vaccine uptake—was not statistically significant. However, when we performed robustness checks with vaccine uptake measured as the share of the population with only one dose and when removing outliers, a positive and statistically significant association between voter turnout and vaccine uptake at the municipality level can be seen. There are at least two potential explanations for the inconclusive results for voter turnout. One is that the correlation between turnout and vaccine uptake is weak, which would provide a potential explanation for the inconsistency between different model specifications. The second explanation is that there is a substantial difference between what factors are associated with imperfect compliance (one dose of the vaccine) compared with full compliance (at the time, two doses of vaccine). This will have to be further studied in future research, perhaps using microlevel data. Similarly, the type of religiosity that we measured is not associated with vaccine uptake. This could indicate that the Evangelically oriented religious communities in Sweden were not skeptical toward vaccines in general in Sweden, or it could be a result of the poor construct validity of the measurement of religiosity.

The policy implications of our findings are somewhat complex. The negative association between the presence of first-generation non-European immigrants and vaccine uptake suggests that special efforts to reach groups with Swedish as a second language are needed. Projects aiming to reach out to groups with Swedish as a second language were organized throughout 2021, and vaccine uptake gradually increased in the majority of immigrant neighborhoods [85].

Our findings indicate that if groups that are likely to have lower levels of trust in government institutions constitute a relatively large share of the population in a municipality, then the likely average vaccine within the local community uptake will be below average. Given this common denominator, we propose further investigating the role of distrust in public institutions for variations in vaccine uptake. Given that our study concerns–a country with high average levels of institutional trust and a long history of successful vaccination programs (i.e., a least likely case of variations in vaccine uptake)–suggests that these policy problems are likely not unique to Sweden and relatively independent of the quality of political and governmental institutions.

However, it remains arguably a difficult task for public agencies to try to convince skeptical citizens who generally distrust public institutions to listen to their recommendations and vaccinate. Recent experiments from Sweden have shown that nudging and information had no significant impact on the willingness to vaccinate [86].

Our results are robust to the introduction of several socioeconomic control variables; however, we would like to point to the possibility of ecological fallacies [87], that is, other unobserved individual-level factors that explain the associations. Further studies using neighborhood-level and individual-level data are needed to disentangle the importance of distrust in government institutions for willingness to vaccinate.

## Supporting information

S1 Fig Scatterplots visualizing the associations between the predicted vaccine uptake and (A) share of SD voters in the 2018 Swedish General Election, (B) Voter turnout in the 2018 General Election, (C) Share of population who are members of a free church, (D) Share of foreign-born, (E) Share of the population born outside Europe, and (F) Share of the population born in Europe (excluding Sweden). (TIFF)

S2 Fig Scatterplot matrix of all included variables in the analyses. (TIFF)

S3 Fig Scatterplot of standardized regression residuals and fitted values. (TIFF)

S4 Fig Normal probability plot of the regression models. (TIFF)

S5 Fig Predicted and observed vaccination rates. (TIFF)

S1 Table Descriptive statistics of all included variables in the analyses. (DOCX)

S2 Table Correlation matrix of all included variables in the analyses. (DOCX)

S3 Table Variance inflation factor (VIF) tests for all included models. (DOCX)

S4 Table OLS regression models without outliers. (DOCX)

S5 Table OLS regression models with partial compliance (1 dose) as the dependent variable. (DOCX)

S6 Table OLS regression models without potentially confounding variables. (DOCX)

S7 Table Beta-regression models. (DOCX)

## References

[1] World Health Organisation. WHO Coronavirus (COVID-19) Dashboard [Internet]. 2022. https://covid19.who.int/

[2] Madhav N, Oppenheim B, Gallivan M, Mulembakani P, Rubin E, Wolfe N. Pandemics: Risks, Impacts, and Mitigation. In: Jamison DT, Gelband H, Horton S, Jha P, Laxminarayan R, Mock CN, et al., editors. Disease Control Priorities: Improving Health and Reducing Poverty [Internet]. 3rd ed. Washington (DC): The International Bank for Reconstruction and Development / The World Bank; 2017 [cited 2021 Jul 8]. http://www.ncbi.nlm.nih.gov/books/NBK525302/

[3] Cauchemez S, Ferguson NM, Wachtel C, Tegnell A, Saour G, Duncan B, et al. Closure of schools during an influenza pandemic. The Lancet Infectious Diseases. 2009 Aug;9(8):473–81. doi: 10.1016/S1473-3099(09)70176-8 19628172 PMC7106429

[4] Osterrieder A, Cuman G, Pan-Ngum W, Cheah PK, Cheah PK, Peerawaranun P, et al. Economic and social impacts of COVID-19 and public health measures: results from an anonymous online survey in Thailand, Malaysia, the UK, Italy and Slovenia. BMJ Open. 2021 Jul 1;11(7):e046863. doi: 10.1136/bmjopen-2020-046863 34285007 PMC8295020

[5] Alexander SA, Shareck M. Widening the gap? Unintended consequences of health promotion measures for young people during COVID-19 lockdown. Health Promot Int [Internet]. 2021 [cited 2021 Oct 13]; https://academic.oup.com/heapro/advance-article/doi/10.1093/heapro/daab015/6144787 33604653 10.1093/heapro/daab015 PMC7928856

[6] Ghazawy ER, Ewis AA, Mahfouz EM, Khalil DM, Arafa A, Mohammed Z, et al. Psychological impacts of COVID-19 pandemic on the university students in Egypt. Health Promot Int. 2021 Aug 30;36(4):1116–25. doi: 10.1093/heapro/daaa147 33367587 PMC7799058

[7] McGrath A, Murphy N, Richardson N. The impact of the COVID-19 pandemic on the wellbeing of Irish Men’s Shed members. Health Promot Int. 2021 Aug 30;36(4):1007–19. doi: 10.1093/heapro/daaa113 33270821 PMC7799116

[8] Wouters OJ, Shadlen KC, Salcher-Konrad M, Pollard AJ, Larson HJ, Teerawattananon Y, et al. Challenges in ensuring global access to COVID-19 vaccines: production, affordability, allocation, and deployment. The Lancet. 2021 Mar 13;397(10278):1023–34. 10.1016/S0140-6736(21)00306-8 PMC7906643 33587887

[9] Mulberry N, Tupper P, Kirwin E, McCabe C, Colijn C. Vaccine rollout strategies: The case for vaccinating essential workers early. de A Pereira CC, editor. PLOS Glob Public Health. 2021 Oct 13;1(10):e0000020. 10.1371/journal.pgph.0000020 PMC10021761 36962089

[10] Anderson RM, Vegvari C, Truscott J, Collyer BS. Challenges in creating herd immunity to SARS-CoV-2 infection by mass vaccination. The Lancet. 2020 Nov 21;396(10263):1614–6. doi: 10.1016/S0140-6736(20)32318-7 33159850 PMC7836302

[11] Mills M, Rahal, Brazel, Yan, Gieysztor. COVID-19 Vaccine Deployment: Behaviour, Ethics, Misinformation and Policy Strategies [Internet]. London: The Royal Society & The British Academy; 2020 [cited 2021 Jul 5]. https://www.socialscienceinaction.org/resources/covid-19-vaccine-deployment-behaviour-ethics-misinformation-and-policy-strategies/

[12] Robertson E, Reeve KS, Niedzwiedz CL, Moore J, Blake M, Green M, et al. Predictors of COVID-19 vaccine hesitancy in the UK household longitudinal study. Brain Behav Immun. 2021 May;94:41–50. doi: 10.1016/j.bbi.2021.03.008 33713824 PMC7946541

[13] Khubchandani J, Sharma S, Price JH, Wiblishauser MJ, Sharma M, Webb FJ. COVID-19 Vaccination Hesitancy in the United States: A Rapid National Assessment. J Community Health. 2021 Apr;46(2):270–7. doi: 10.1007/s10900-020-00958-x 33389421 PMC7778842

[14] Kennedy J. Populist politics and vaccine hesitancy in Western Europe: an analysis of national-level data. European Journal of Public Health. 2019 Jun 1;29(3):512–6. doi: 10.1093/eurpub/ckz004 30801109

[15] Jama A, Ali M, Lindstrand A, Butler R, Kulane A. Perspectives on the Measles, Mumps and Rubella Vaccination among Somali Mothers in Stockholm. International Journal of Environmental Research and Public Health. 2018 Nov;15(11):2428. doi: 10.3390/ijerph15112428 30388799 PMC6265853

[16] Byström E, Lindstrand A, Likhite N, Butler R, Emmelin M. Parental attitudes and decision-making regarding MMR vaccination in an anthroposophic community in Sweden–A qualitative study. Vaccine. 2014 Nov 28;32(50):6752–7. doi: 10.1016/j.vaccine.2014.10.011 25454859

[17] Feikin DR, Lezotte D, Hamman R, Salmon DA, Chen R, Hoffman R. Individual and Community Risks of Measles and Pertussis Associated With Personal Exemptions to Immunization. JAMA. 2000 Dec 27;284(24):3145. doi: 10.1001/jama.284.24.3145 11135778

[18] Omer SB, Salmon DA, Orenstein WA, deHart MP, Halsey N. Vaccine refusal, mandatory immunization, and the risks of vaccine-preventable diseases. N Engl J Med. 2009 May 7;360(19):1981–8. doi: 10.1056/NEJMsa0806477 19420367

[19] Sköld P. From inoculation to vaccination: Smallpox in Sweden in the eighteenth and nineteenth centuries. Population Studies. 1996;50(2):247–62. doi: 10.1080/0032472031000149336 11613334

[20] Snyder R. Scaling down: The subnational comparative method. Studies in comparative international development. 2001;36(1):93–110.

[21] Brandén M, Aradhya S, Kolk M, Härkönen J, Drefahl S, Malmberg B, et al. Residential context and COVID-19 mortality among adults aged 70 years and older in Stockholm: a population-based, observational study using individual-level data. The Lancet Healthy Longevity. 2020 Nov 1;1(2):e80–8. doi: 10.1016/S2666-7568(20)30016-7 33521770 PMC7832817

[22] Lassale C, Gaye B, Hamer M, Gale CR, Batty GD. Ethnic disparities in hospitalisation for COVID-19 in England: The role of socioeconomic factors, mental health, and inflammatory and pro-inflammatory factors in a community-based cohort study. Brain, Behavior, and Immunity. 2020 Aug;88:44–9. doi: 10.1016/j.bbi.2020.05.074 32497776 PMC7263214

[23] Bromme R, Mede NG, Thomm E, Kremer B, Ziegler R. An anchor in troubled times: Trust in science before and within the COVID-19 pandemic. PLOS ONE. 2022 Feb 9;17(2):e0262823. doi: 10.1371/journal.pone.0262823 35139103 PMC8827432

[24] Ward JK, Alleaume C, Peretti-Watel P, Peretti-Watel P, Seror V, Cortaredona S, et al. The French public’s attitudes to a future COVID-19 vaccine: The politicization of a public health issue. Social Science & Medicine. 2020 Nov 1;265:113414. doi: 10.1016/j.socscimed.2020.113414 33038683 PMC7537647

[25] Folkhälsomyndigheten. Risk för intensivvård med covid-19 i olika typer av områden [Internet]. 2022. Report No.: 22144. https://www.folkhalsomyndigheten.se/publicerat-material/publikationsarkiv/r/risk-for-intensivvard-med-covid-19-i-olika-typer-av-omraden/?

[26] Villa S, Lombardi A, Mangioni D, Bozzi G, Bandera A, Gori A, et al. The COVID-19 pandemic preparedness … or lack thereof: from China to Italy. Global Health & Medicine. 2020;2(2):73–7. 33330781 10.35772/ghm.2020.01016 PMC7731270

[27] Ross AGP, Crowe SM, Tyndall MW. Planning for the Next Global Pandemic. International Journal of Infectious Diseases. 2015 Sep 1;38:89–94. doi: 10.1016/j.ijid.2015.07.016 26253461 PMC7128994

[28] Epstein JM. Are We Already Missing the Next Epidemic. Politico [Internet]. 2020 Mar 31; https://www.politico.com/news/magazine/2020/03/31/coronavirus-americafear-contagion-can-we-handle-it-157711

[29] Chua AQ, Knawy BA, Grant B, Legido-Quigley H, Lee WC, Leung GM, et al. How the lessons of previous epidemics helped successful countries fight covid-19. BMJ. 2021 Mar 11;372:n486. doi: 10.1136/bmj.n486 33707174

[30] MacDonald NE. Vaccine hesitancy: Definition, scope and determinants. Vaccine. 2015 Aug 14;33(34):4161–4. doi: 10.1016/j.vaccine.2015.04.036 25896383

[31] Truong J, Bakshi S, Wasim A, Ahmad M, Majid U. What factors promote vaccine hesitancy or acceptance during pandemics? A systematic review and thematic analysis. Health Promotion International [Internet]. 2021 Jul 9 [cited 2021 Oct 13];(daab105). 10.1093/heapro/daab105 34244738

[32] Crawshaw AF, Farah Y, Deal A, Rustage K, Hayward SE, Carter J, et al. Defining the determinants of vaccine uptake and undervaccination in migrant populations in Europe to improve routine and COVID-19 vaccine uptake: a systematic review. The Lancet Infectious Diseases. 2022 Apr;S1473309922000664. doi: 10.1016/S1473-3099(22)00066-4 35429463 PMC9007555

[33] Wollebæk D, Fladmoe A, Steen‐Johnsen K, Ihlen Ø. Right‐wing ideological constraint and vaccine refusal: The case of the COVID‐19 vaccine in Norway. Scand Pol Stud. 2022 Jun;45(2):253–78. doi: 10.1111/1467-9477.12224 35600113 PMC9111158

[34] Jennings W, Stoker G, Willis H, Valgardsson V, Gaskell J, Devine D, et al. Lack of trust and social media echo chambers predict COVID-19 vaccine hesitancy. In: medRxiv [Internet]. Cold Spring Harbor Laboratory Press; 2021 [cited 2021 Jul 5]. p. 2021.01.26.21250246. https://www.medrxiv.org/content/10.1101/2021.01.26.21250246v1

[35] Folkhälsomyndigheten. Acceptans för covid-19-vaccination–Invånare i Sverige födda i Afrika och Mellanöstern [Internet]. 2021. https://www.folkhalsomyndigheten.se/publicerat-material/publikationsarkiv/a/acceptans-for-covid-19-vaccination-del2/#:~:text=Unders%C3%B6kningen%20genomf%C3%B6rdes%20under%20augusti%20och,till%20vaccination%20mot%20covid%2D19.

[36] Barrios JM, Benmelech E, Hochberg YV, Sapienza P, Zingales L. Civic capital and social distancing during the Covid-19 pandemic☆. Journal of Public Economics. 2021 Jan;193:104310. 33199928 10.1016/j.jpubeco.2020.104310 PMC7657101

[37] Lindholt MF, Jørgensen F, Bor A, Petersen MB. Public acceptance of COVID-19 vaccines: cross-national evidence on levels and individual-level predictors using observational data. BMJ Open. 2021 Jun;11(6):e048172. doi: 10.1136/bmjopen-2020-048172 34130963 PMC8210695

[38] Akel KB, Noppert GA, Rajamoorthy Y, Lu Y, Singh A, Harapan H, et al. A study of COVID-19 vaccination in the US and Asia: The role of media, personal experiences, and risk perceptions. PLOS Global Public Health. 2022 Jul 13;2(7):e0000734. 10.1371/journal.pgph.0000734 PMC10021344 36962371

[39] Romer D, Jamieson KH. Conspiracy theories as barriers to controlling the spread of COVID-19 in the U.S. Social Science & Medicine. 2020 Oct 1;263:113356. doi: 10.1016/j.socscimed.2020.113356 32967786 PMC7502362

[40] Amit AML, Pepito VCF, Sumpaico-Tanchanco L, Dayrit MM. COVID-19 vaccine brand hesitancy and other challenges to vaccination in the Philippines. PLOS Global Public Health. 2022 Jan 13;2(1):e0000165. 10.1371/journal.pgph.0000165 PMC10021706 36962166

[41] Karlsson LC, Soveri A, Lewandowsky S, Karlsson L, Karlsson H, Nolvi S, et al. Fearing the disease or the vaccine: The case of COVID-19. Personality and Individual Differences. 2021 Apr;172:110590. doi: 10.1016/j.paid.2020.110590 33518869 PMC7832025

[42] Barda N, Dagan N, Ben-Shlomo Y, Kepten E, Waxman J, Ohana R, et al. Safety of the BNT162b2 mRNA Covid-19 Vaccine in a Nationwide Setting. N Engl J Med. 2021 Sep 16;385(12):1078–90. doi: 10.1056/NEJMoa2110475 34432976 PMC8427535

[43] Kranz D, Niepel C, Botes E, Greiff S. Religiosity Predicts Unreasonable Coping With COVID-19. Psychology of Religion and Spirituality. 2020.

[44] Lucia VC, Kelekar A, Afonso NM. COVID-19 vaccine hesitancy among medical students. J Public Health (Oxf). 2021 Sep 22;43(3):445–9. 33367857 10.1093/pubmed/fdaa230 PMC7799040

[45] Latkin CA, Dayton L, Yi G, Konstantopoulos A, Boodram B. Trust in a COVID-19 vaccine in the U.S.: A social-ecological perspective. Soc Sci Med. 2021 Feb;270:113684. doi: 10.1016/j.socscimed.2021.113684 33485008 PMC7834519

[46] Baumgaertner B, Carlisle JE, Justwan F. The influence of political ideology and trust on willingness to vaccinate. Rabinowitz M, editor. PLoS ONE. 2018 Jan 25;13(1):e0191728. doi: 10.1371/journal.pone.0191728 29370265 PMC5784985

[47] Yuan H, Long Q, Huang G, Huang L, Luo S. Different roles of interpersonal trust and institutional trust in COVID-19 pandemic control. Social Science & Medicine. 2021 Dec;114677. doi: 10.1016/j.socscimed.2021.114677 35101260 PMC8692240

[48] Khan T, Nassrally S, Nassrally S, Guy’s and St Thomas’ Hospital, London. Is fake news contributing to increased Covid-19 BAME deaths? Acute Med J. 2020 Apr 1;19(2):110–110. 32840263

[49] de Figueiredo A, Simas C, Karafillakis E, Paterson P, Larson HJ. Mapping global trends in vaccine confidence and investigating barriers to vaccine uptake: a large-scale retrospective temporal modelling study. The Lancet. 2020 Sep 26;396(10255):898–908. doi: 10.1016/S0140-6736(20)31558-0 32919524 PMC7607345

[50] Grönlund K, Setälä M. Political trust, satisfaction and voter turnout. Comparative European Politics. 2007;5(4):400–22.

[51] Andersson U, Oscarsson H. Institutionsförtroendet inte lika politiserat under pandemin. SOM-Undersökningen om Coronaviruset. 2020.

[52] Widfeldt A. The radical right in the Nordic Countries. In: The Oxford handbook of the radical right. 2018.

[53] De Lange SL, Mudde C. Political extremism in Europe. Eur Polit Sci. 2005 Dec;4(4):476–88.

[54] Bonikowski B, Halikiopoulou D, Kaufmann E, Rooduijn M. Populism and nationalism in a comparative perspective: a scholarly exchange. Nations and Nationalism. 2019;25(1):58–81.

[55] Jylhä K, Rydgren J, Strimling P. Sverigedemokraternas väljare. Vilka är de, var kommer de ifrån och vart är de på väg. Institutet för Framtidsstudier. 2018;1–100.

[56] Mudde C. The populist radical right: A pathological normalcy. west european politics. 2010;33(6):1167–86.

[57] Mudde C. Three decades of populist radical right parties in Western Europe: So what? European Journal of Political Research. 2013;52(1):1–19.

[58] Mudde C. The populist zeitgeist. Government and opposition. 2004;39(4):541–63.

[59] Rooduijn M, van Kessel S, Froio C, Pirro A, De Lange SL, Halikiopoulou D, et al. PopuList: An overview of populist, far right, far left and Eurosceptic parties in Europe. [Internet]. 2019. www.popu-list.org.

[60] Esaiasson P, Sohlberg J, Ghersetti M, Johansson B. How the coronavirus crisis affects citizen trust in institutions and in unknown others: Evidence from ‘the Swedish experiment’. European Journal of Political Research. 2021;60(3):748–60.

[61] VoF [Föreningen för Vetenskap och Fortbildning]. VoF-undersökningen 2021: Konspirationsteorier, vidskepelse och pseudovetenskap i Sverige. 2021.

[62] Swedish Radio. Ny mätning: Vaccinationsviljan mindre bland SD-väljare. Nyheter (Ekot). 2021 0121.

[63] Johnson JH, Bonds JM, Parnell AM, Bright CM. Coronavirus Vaccine Distribution: Moving to a Race Conscious Approach for a Racially Disparate Problem. J Racial Ethn Health Disparities. 2021 Aug;8(4):799–802. doi: 10.1007/s40615-021-01051-2 33948908 PMC8095217

[64] Callaghan T, Moghtaderi A, Lueck JA, Hotez P, Strych U, Dor A, et al. Correlates and disparities of intention to vaccinate against COVID-19. Soc Sci Med. 2021 Mar;272:113638. doi: 10.1016/j.socscimed.2020.113638 33414032 PMC7834845

[65] Díaz E, Norredam M, Aradhya S, Benfield T, Krasnik A, Madar A, et al. Situational Brief: Migration and COVID-19 in Scandinavian Countries. Lancet Migration. 2020 Dec 18.

[66] Rothstein B, Holmberg S. Den svenska tilliten fortsatt hög–men sjunker i utsatta grupper. In: Du sköra nya värld. Göteborg: SOM-institutet, Göteborgs universitet.; 2021.

[67] Andersson R, Hedman L. Economic decline and residential segregation: a Swedish study with focus on Malmö. Urban Geography. 2016 Jul 3;37(5):748–68.

[68] de Vroome T, Hooghe M, Marien S. The Origins of Generalized and Political Trust among Immigrant Minorities and the Majority Population in the Netherlands. European Sociological Review. 2013 Dec;29(6):1336–50.

[69] Hero RE, Tolbert CJ. Minority voices and citizen attitudes about government responsiveness in the American states: Do social and institutional context matter? British Journal of Political Science. 2004;34(1):109–21.

[70] Garcia LL, Yap JFC. The role of religiosity in COVID-19 vaccine hesitancy. J Public Health (Oxf). 2021 Jun 3;fdab192. doi: 10.1093/pubmed/fdab192 34080617 PMC8195070

[71] Olagoke AA, Olagoke OO, Hughes AM. Intention to Vaccinate Against the Novel 2019 Coronavirus Disease: The Role of Health Locus of Control and Religiosity. J Relig Health. 2021 Feb;60(1):65–80. doi: 10.1007/s10943-020-01090-9 33125543 PMC7596314

[72] Wallman Lundåsen S. Religious Participation and Civic Engagement in a Secular Context: Evidence from Sweden on the Correlates of Attending Religious Services. Voluntas [Internet]. 2021 May 4 [cited 2021 Oct 19]; https://link.springer.com/10.1007/s11266-021-00353-7

[73] Lundåsen SW, Trägårdh L. Social trust and religion in Sweden: Theological belief versus social organization. In: Religion and civil society in Europe. Springer; 2013. p. 109–24.

[74] European Medicines Agency. COVID-19 vaccine safety update, 18 June 2021. COMIRNATY [Internet]. 2021. https://www.ema.europa.eu/en/documents/covid-19-vaccine-safety-update/covid-19-vaccine-safety-update-comirnaty-18-june-2021_en.pdf

[75] European Medicines Agency. COVID-19 vaccine safety update, 18 June 2021. COVID-19 Vaccine Moderna [Internet]. 2021. https://www.ema.europa.eu/en/documents/covid-19-vaccine-safety-update/covid-19-vaccine-safety-update-spikevax-previously-covid-19-vaccine-moderna-18-june-2021_en.pdf

[76] European Medicines Agency. COVID-19 vaccine safety update, 18 June 2021. Vaxzevria. [Internet]. 2021. https://www.ema.europa.eu/en/documents/covid-19-vaccine-safety-update/covid-19-vaccine-safety-update-vaxzevria-previously-covid-19-vaccine-astrazeneca-18-june-2021_en.pdf

[77] Swedish Public Health Agency. Statistik för vaccination mot covid-19 [Internet]. 2022. https://www.folkhalsomyndigheten.se/folkhalsorapportering-statistik/statistikdatabaser-och-visualisering/vaccinationsstatistik/statistik-for-vaccination-mot-covid-19/

[78] Statistics Sweden. Statistikdatabasen [Internet]. 2021. https://www.statistikdatabasen.scb.se/pxweb/sv/ssd/

[79] Tholvsen Ø. Frikyrkounderso kningen—En rapport om frikyrkornas utveckling i Sverige 2000–2020. Sveriges Frikyrkosamråd; 2021.

[80] Swedish Association of Local Authorities and Regions. Kolada [Internet]. Kolada. 2021. www.kolada.se

[81] Ferrari A, Comelli M. A comparison of methods for the analysis of binomial clustered outcomes in behavioral research. Journal of Neuroscience Methods. 2016 Dec 1;274:131–40. doi: 10.1016/j.jneumeth.2016.10.005 27751892

[82] Ferrari S, Cribari-Neto F. Beta Regression for Modelling Rates and Proportions. Journal of Applied Statistics. 2004 Aug 1;31(7):799–815.

[83] Böttiger M, Christenson B, Romanus V, Taranger J, Strandell A. Swedish experience of two dose vaccination programme aiming at eliminating measles, mumps, and rubella. Br Med J (Clin Res Ed). 1987 Nov 14;295(6608):1264–7. 10.1136/bmj.295.6608.1264 PMC1248321 3120971

[84] Burström B. Social Differentials in the Decline of Infant Mortality in Sweden in the Twentieth Century: The Impact of Politics and Policy. Int J Health Serv. 2003 Oktober;33(4):723–41. doi: 10.2190/9GMR-TA8W-LA3B-5E2A 14758857

[85] Dagens Nyheter. Fakta i frågan: Vilka är de ovaccinerade? [Internet]. DN.SE. 2021 [cited 2022 Feb 18]. https://www.dn.se/sverige/fakta-i-fragan-vilka-ar-de-ovaccinerade/

[86] Campos-Mercade P, Meier AN, Schneider FH, Meier S, Pope D, Wengström E. Monetary incentives increase COVID-19 vaccinations. Science. 2021;374(6569):879–82. doi: 10.1126/science.abm0475 34618594 PMC10765478

[87] Robinson W. Ecological Correlations and the Behavior of Individuals. International Journal of Epidemiology. 2009 Apr;38(2):337–41. doi: 10.1093/ije/dyn357 19179346

