# Correction: Variations in vaccination uptake: COVID-19 vaccination rates in Swedish municipalities

**DOI:** 10.1371/journal.pgph.0003397

**Published:** 2024-06-13

**Authors:** The PLOS Global Public Health Staff

The author initials appear incorrectly in the citation. The correct citation is: Carlberg Larsson E, Wittberg E, Wallman Lundåsen S (2022). Variations in vaccination uptake: COVID-19 vaccination rates in Swedish municipalities. PLOS Glob Public Health 2(10): e0001204. https://doi.org/10.1371/journal.pgph.0001204

The publisher apologizes for the error.
