# Supplementary figures and images for: Variations in vaccination uptake: COVID-19 vaccination rates in Swedish municipalities
Source: PLOS Glob Public Health. 2022 Oct 20;2(10):e0001204. doi: 10.1371/journal.pgph.0001204 (PMC10022166; doi:10.1371/journal.pgph.0001204)

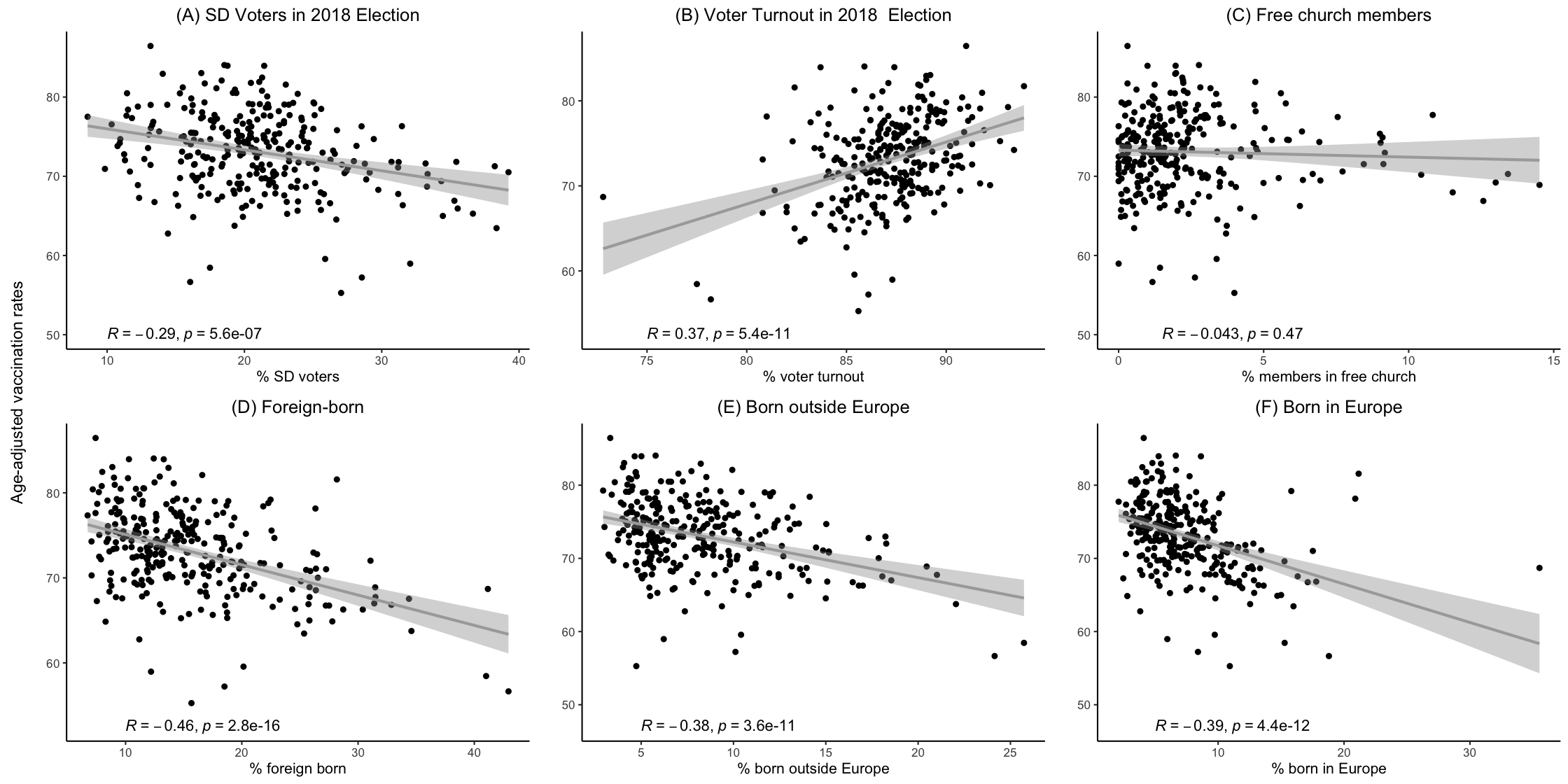

Supplement: S1 Fig — (TIFF) [file pgph.0001204.s001.tiff]

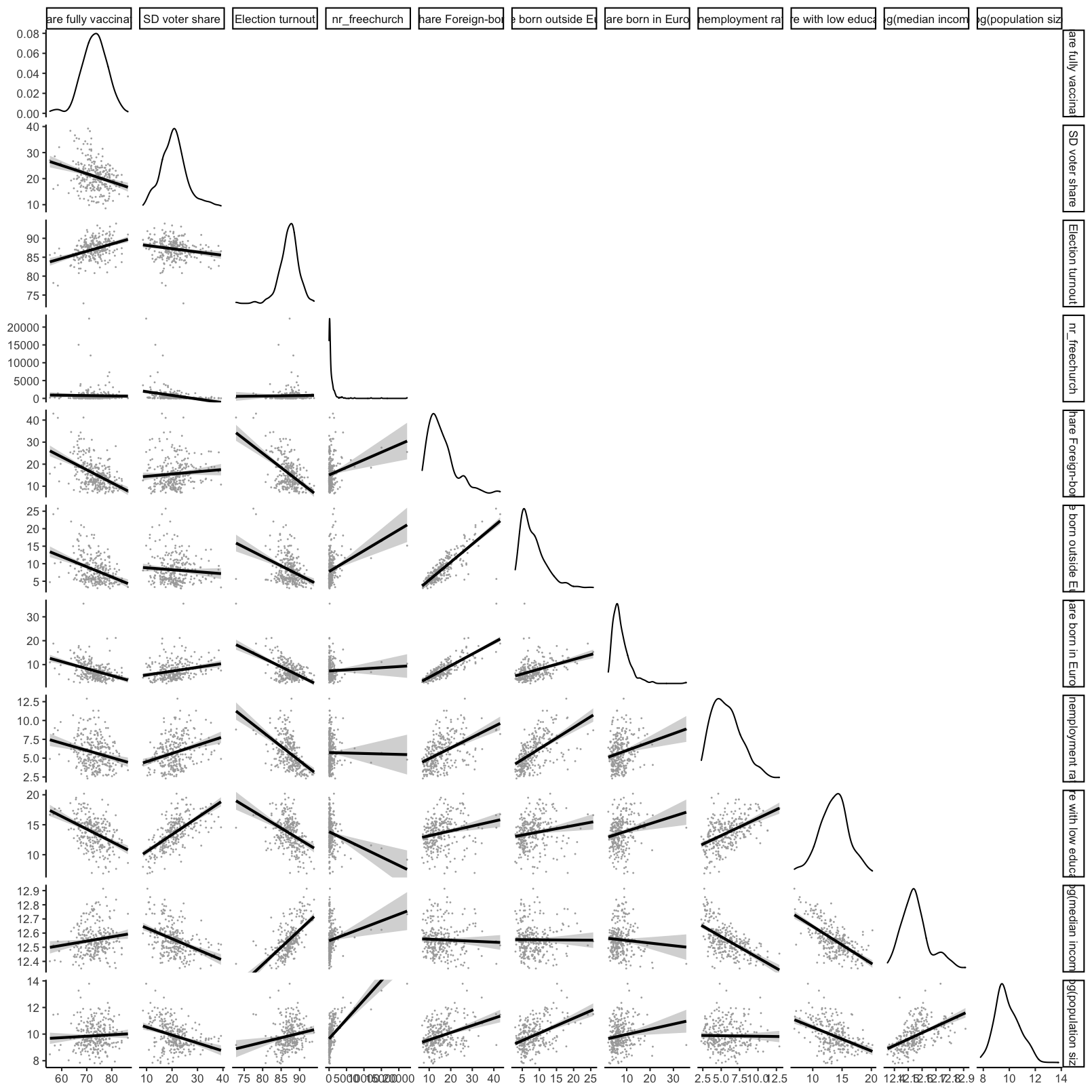

Supplement: S2 Fig — (TIFF) [file pgph.0001204.s002.tiff]

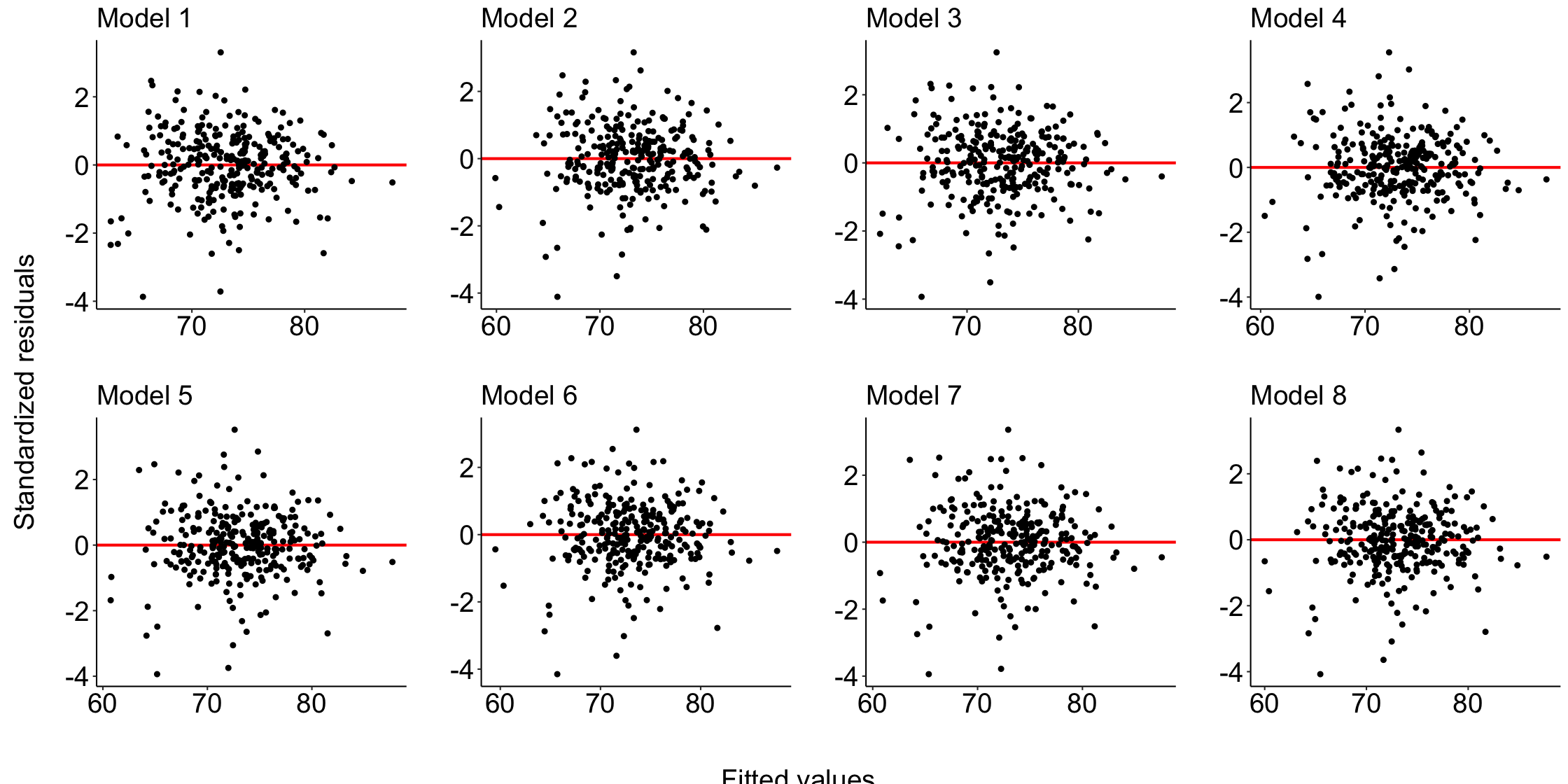

Supplement: S3 Fig — (TIFF) [file pgph.0001204.s003.tiff]

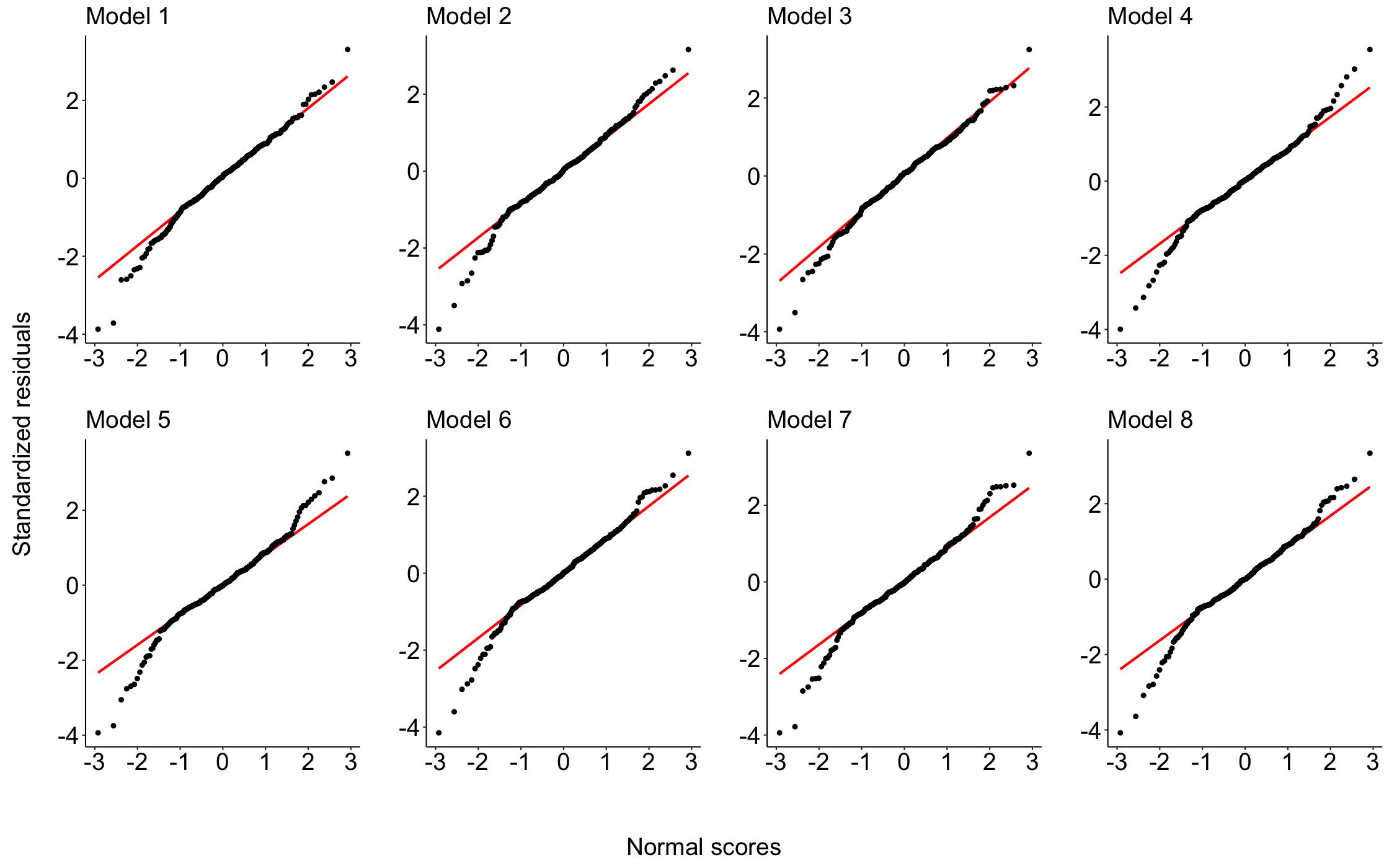

Supplement: S4 Fig — (TIFF) [file pgph.0001204.s004.tiff]

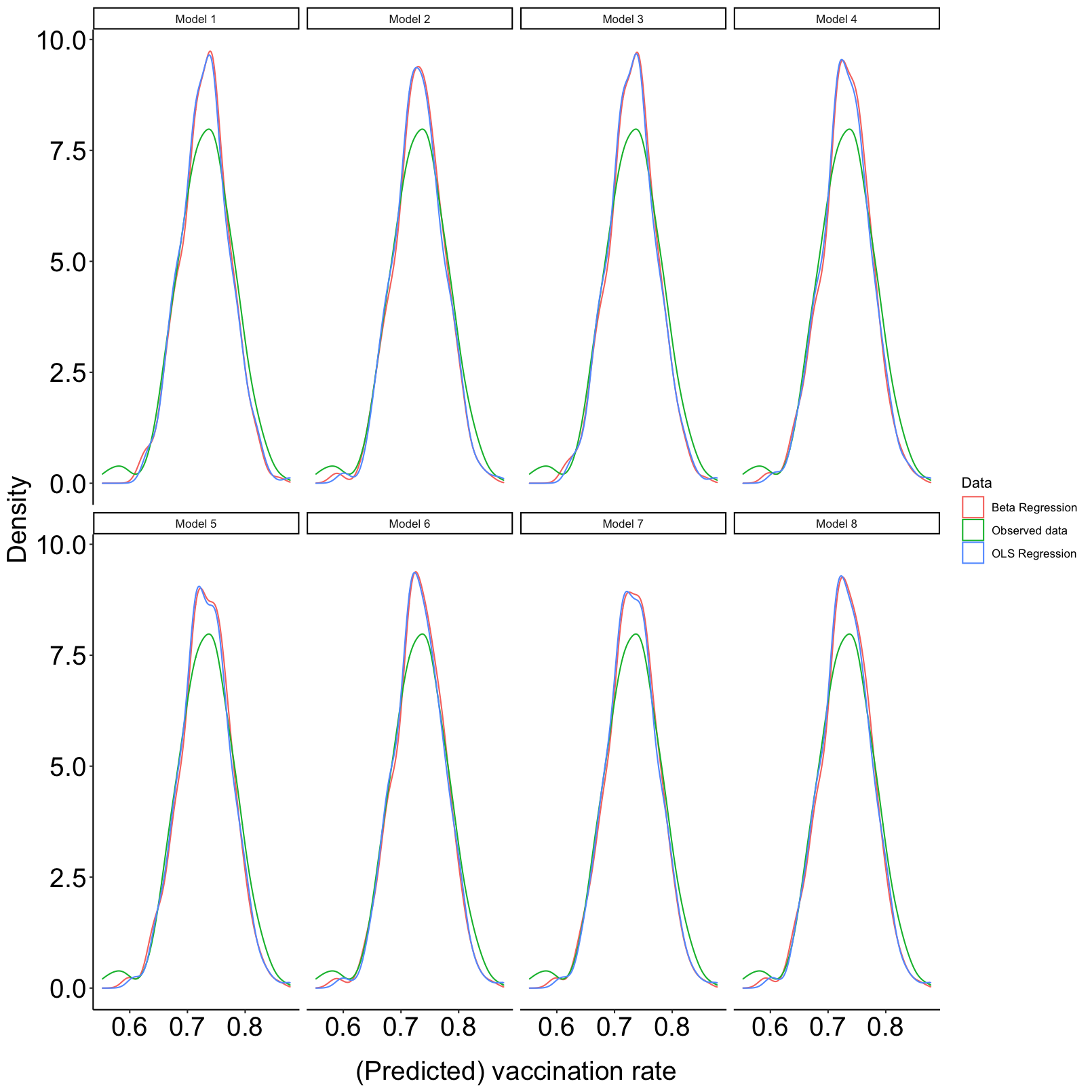

Supplement: S5 Fig — (TIFF) [file pgph.0001204.s005.tiff]
