# Supplementary material for: Variations in vaccination uptake: COVID-19 vaccination rates in Swedish municipalities
Source: PLOS Glob Public Health. 2022 Oct 20;2(10):e0001204. doi: 10.1371/journal.pgph.0001204 (PMC10022166; doi:10.1371/journal.pgph.0001204)
Supplement: S1 Table — (DOCX) [file pgph.0001204.s006.docx]

**S1 Table.** Descriptive statistics of all included variables in the analyses.

|  | **N** | **Mean** | **St. Dev.** | **Min** | **Pctl(25)** | **Pctl(75)** | **Max** |
| --- | --- | --- | --- | --- | --- | --- | --- |
| Age-standardized vaccination rate | 290 | 73.09 | 4.98 | 55 | 70.0 | 76.4 | 86 |
| SD voter share | 290 | 20.93 | 5.43 | 8.58 | 17.32 | 23.51 | 39.23 |
| Election turnout | 290 | 87.18 | 2.54 | 72.80 | 85.82 | 88.60 | 93.90 |
| Members in free church | 290 | 2.31 | 2.43 | 0.00 | 0.78 | 2.82 | 14.51 |
| Share Foreign-born | 290 | 15.62 | 6.37 | 6.73 | 11.18 | 18.50 | 42.94 |
| Share born outside Europe | 290 | 8.22 | 3.85 | 2.94 | 5.45 | 10.00 | 25.73 |
| Share born in Europe | 290 | 7.40 | 3.72 | 2.10 | 4.96 | 8.89 | 35.52 |
| Unemployment rate | 290 | 5.73 | 1.98 | 2.30 | 4.12 | 7.00 | 12.90 |
| Share with low education | 290 | 13.62 | 2.43 | 6.87 | 11.97 | 15.16 | 20.18 |
| Log(median income) | 290 | 12.55 | 0.10 | 12.35 | 12.49 | 12.60 | 12.91 |
| Log(population size) | 290 | 9.87 | 0.98 | 7.79 | 9.22 | 10.49 | 13.79 |
