# Supplementary material for: Variations in vaccination uptake: COVID-19 vaccination rates in Swedish municipalities
Source: PLOS Glob Public Health. 2022 Oct 20;2(10):e0001204. doi: 10.1371/journal.pgph.0001204 (PMC10022166; doi:10.1371/journal.pgph.0001204)
Supplement: S2 Table — (DOCX) [file pgph.0001204.s007.docx]

**S2 Table.** Correlation matrix of all included variables in the analyses.

|  | 1 | 2 | 3 | 4 | 5 | 6 | 7 | 8 | 9 | 10 | 11 |
| --- | --- | --- | --- | --- | --- | --- | --- | --- | --- | --- | --- |
| 1. Age-standardized vaccination rate | 1 |  |  |  |  |  |  |  |  |  |  |
| 2. SD voter share | - 0.29 | 1 |  |  |  |  |  |  |  |  |  |
| 3. Election turnout | 0.37 | - 0.19 | 1 |  |  |  |  |  |  |  |  |
| 4. Members in free church | - 0.04 | - 0.16 | 0.05 | 1 |  |  |  |  |  |  |  |
| 5. Share Foreign-born | - 0.46 | 0.09 | - 0.52 | - 0.11 | 1 |  |  |  |  |  |  |
| 6. Share born outside Europe | - 0.38 | - 0.08 | - 0.35 | - 0.02 | 0.85 | 1 |  |  |  |  |  |
| 7. Share born in Europe | - 0.39 | 0.23 | - 0.52 | - 0.16 | 0.84 | 0.42 | 1 |  |  |  |  |
| 8. Unemployment rate | - 0.24 | 0.30 | - 0.49 | - 0.07 | 0.46 | 0.55 | 0.21 | 1 |  |  |  |
| 9. Share with low education | - 0.43 | 0.63 | - 0.39 | 0.14 | 0.21 | 0.17 | 0.19 | 0.47 | 1 |  |  |
| 10. Log(median income) | 0.15 | - 0.40 | 0.61 | - 0.06 | -0.04 | - 0.01 | -0.07 | - 0.58 | -0.62 | 1 |  |
| 11. Log(population size) | 0.05 | - 0.33 | 0.18 | - 0.14 | 0.35 | 0.44 | 0.15 | - 0.02 | -0.44 | 0.50 | 1 |
