# Supplementary material for: Variations in vaccination uptake: COVID-19 vaccination rates in Swedish municipalities
Source: PLOS Glob Public Health. 2022 Oct 20;2(10):e0001204. doi: 10.1371/journal.pgph.0001204 (PMC10022166; doi:10.1371/journal.pgph.0001204)
Supplement: S3 Table — (DOCX) [file pgph.0001204.s008.docx]

**S3 Table.** Variance inflation factor (VIF) tests for all included models

|  | **Model 1** | **Model 2** | **Model 3** | **Model 4** | **Model 5** | **Model 6** | **Model 7** | **Model 8** |
| --- | --- | --- | --- | --- | --- | --- | --- | --- |
| SD voter share | 4.634 |  |  |  | 5.113 | 5.294 | 4.828 | 5.317 |
| Election turnout |  | 2.993 |  |  | 5.892 | 3.612 | 5.358 | 6.027 |
| Members in free church |  |  | 2.075 |  | 2.194 | 2.216 | 2.189 |  |
| Share Foreign-born |  |  |  | 2.904 | 5.786 |  |  |  |
| Share born outside Europe |  |  |  |  |  | 4.993 |  | 5.002 |
| Share born in Europe |  |  |  |  |  |  | 3.181 | 3.187 |
| Unemployment rate | 2.547 | 2.565 | 2.567 | 2.984 | 3.223 | 4.509 | 2.603 | 4.514 |
| Log(median income) | 4.157 | 5.183 | 4.156 | 4.215 | 5.675 | 5.553 | 5.388 | 5.711 |
| Log(population size) | 2.388 | 2.432 | 2.177 | 2.595 | 2.752 | 2.969 | 2.678 | 2.971 |
| Share with low education | 4.617 | 3.720 | 3.369 | 3.520 | 5.164 | 5.289 | 5.096 | 5.289 |
