# Supplementary material for: Variations in vaccination uptake: COVID-19 vaccination rates in Swedish municipalities
Source: PLOS Glob Public Health. 2022 Oct 20;2(10):e0001204. doi: 10.1371/journal.pgph.0001204 (PMC10022166; doi:10.1371/journal.pgph.0001204)
Supplement: S4 Table — (DOCX) [file pgph.0001204.s009.docx]

**S4 Table.** OLS-regression models without outliers.

|  | | | | | | | | |
| --- | --- | --- | --- | --- | --- | --- | --- | --- |
|  | **Model 1** | **Model 2** | **Model 3** | **Model 4** | **Model 5** | **Model 6** | **Model 7** | **Model 8** |
| SD voter share | - 0.115 |  |  |  | - 0.143^*^ | - 0.161^**^ | - 0.102 | - 0.166^**^ |
|  | (0.061) |  |  |  | (0.061) | (0.061) | (0.060) | (0.061) |
| Election turnout |  | 0.552^***^ |  |  | 0.255 | 0.407^***^ | 0.466^***^ | 0.316^*^ |
|  |  | (0.102) |  |  | (0.140) | (0.108) | (0.138) | (0.141) |
| Members in free church |  |  | 0.004 |  | - 0.072 | - 0.049 | - 0.089 | - 0.051 |
|  |  |  | (0.091) |  | (0.087) | (0.087) | (0.088) | (0.087) |
| Share Foreign-born |  |  |  | - 0.217^***^ | - 0.165^**^ |  |  |  |
|  |  |  |  | (0.039) | (0.054) |  |  |  |
| Share born outside Europe |  |  |  |  |  | - 0.303^***^ |  | - 0.304^***^ |
|  |  |  |  |  |  | (0.082) |  | (0.082) |
| Share born in Europe |  |  |  |  |  |  | - 0.064 | - 0.069 |
|  |  |  |  |  |  |  | (0.071) | (0.069) |
| **Control variables** |  |  |  |  |  |  |  |  |
| Unemployment rate | - 0.194 | - 0.130 | - 0.189 | 0.087 | 0.033 | 0.226 | - 0.154 | 0.217 |
|  | (0.124) | (0.119) | (0.125) | (0.128) | (0.131) | (0.154) | (0.120) | (0.154) |
| Log(median income) | 5.093 | -3.234 | 4.744 | 2.819 | - 0.062 | - 0.309 | -2.452 | 0.321 |
|  | (3.097) | (3.295) | (3.115) | (2.961) | (3.400) | (3.319) | (3.375) | (3.379) |
| Log(population size) | - 0.550^*^ | - 0.063 | - 0.425 | 0.073 | - 0.027 | 0.121 | - 0.166 | 0.113 |
|  | (0.242) | (0.231) | (0.234) | (0.238) | (0.241) | (0.248) | (0.242) | (0.248) |
| Share with low education | - 0.768^***^ | - 0.728^***^ | - 0.905^***^ | - 0.785^***^ | - 0.555^***^ | - 0.520^***^ | - 0.603^***^ | - 0.518^***^ |
|  | (0.137) | (0.115) | (0.117) | (0.112) | (0.134) | (0.134) | (0.135) | (0.134) |
| Constant | 23.014 | 72.504 | 25.787 | 47.216 | 61.279 | 48.494 | 72.303 | 49.376 |
|  | (39.784) | (38.881) | (40.050) | (38.018) | (38.434) | (38.510) | (38.949) | (38.521) |
| Observations | 283 | 283 | 283 | 283 | 283 | 283 | 283 | 283 |
| R^2^ | 0.692 | 0.720 | 0.688 | 0.721 | 0.733 | 0.737 | 0.724 | 0.738 |

***Notes:*** *We performed the equivalent regression models as presented in the article when removing outliers. Outliers are defined as observations whose age-standardized share are outside the inner quartile range. Seven observations are dropped as a result of this definition****^[[1]](#footnote-1)^****.* Unstandardized coefficients; robust standard errors within parentheses. Significance: *p < 0.05; **p < 0.01; ***p < 0.001. All models include county-fixed effects.

1. The following municipalities were identified as outliers: Botkyrka, Södertälje, Dals-Ed, Färelanda, Bengtsfors, Mellerud, and Hammarö. [↑](#footnote-ref-1)
