# Supplementary material for: Variations in vaccination uptake: COVID-19 vaccination rates in Swedish municipalities
Source: PLOS Glob Public Health. 2022 Oct 20;2(10):e0001204. doi: 10.1371/journal.pgph.0001204 (PMC10022166; doi:10.1371/journal.pgph.0001204)
Supplement: S5 Table — (DOCX) [file pgph.0001204.s010.docx]

**S5 Table.** OLS-regression models with partly compliance (1 dose) as dependent variable.

|  | **Model 1** | **Model 2** | **Model 3** | **Model 4** | **Model 5** | **Model 6** | **Model 7** | **Model 8** |
| --- | --- | --- | --- | --- | --- | --- | --- | --- |
| SD voter share | - 0.116^*^ |  |  |  | - 0.158^***^ | - 0.181^***^ | - 0.113^**^ | - 0.186^***^ |
|  | (0.050) |  |  |  | (0.043) | (0.043) | (0.043) | (0.043) |
| Election turnout |  | 0.748^***^ |  |  | 0.437^***^ | 0.588^***^ | 0.668^***^ | 0.486^***^ |
|  |  | (0.073) |  |  | (0.098) | (0.075) | (0.097) | (0.097) |
| Members in free church |  |  | 0.027 |  | - 0.072 | - 0.049 | - 0.090 | - 0.051 |
|  |  |  | (0.075) |  | (0.062) | (0.062) | (0.065) | (0.062) |
| Share Foreign-born |  |  |  | - 0.279^***^ | - 0.180^***^ |  |  |  |
|  |  |  |  | (0.029) | (0.039) |  |  |  |
| Share born outside Europe |  |  |  |  |  | - 0.325^***^ |  | - 0.329^***^ |
|  |  |  |  |  |  | (0.058) |  | (0.058) |
| Share born in Europe |  |  |  |  |  |  | - 0.069 | - 0.080 |
|  |  |  |  |  |  |  | (0.051) | (0.048) |
| **Control variables** |  |  |  |  |  |  |  |  |
| Unemployment rate | - 0.227^*^ | - 0.145 | - 0.218^*^ | .127 | .032 | .232^*^ | - 0.169 | 0.225^*^ |
|  | (0.101) | (0.087) | (0.103) | (0.095) | (0.093) | (0.108) | (0.086) | (0.108) |
| Log(median income) | 8.009^**^ | -3.264 | 7.760^**^ | 5.012^*^ | - 0.162 | - 0.190 | -2.687 | 0.459 |
|  | (2.507) | (2.392) | (2.532) | (2.199) | (2.385) | (2.323) | (2.411) | (2.347) |
|  |  |  |  |  |  |  |  |  |
| Log(population size) | -0.838^***^ | -0.127 | -0.704^***^ | -0.007 | -0.097 | 0.068 | -0.252 | 0.060 |
|  | (0.197) | (0.170) | (0.190) | (0.179) | (0.173) | (0.176) | (0.177) | (0.176) |
| Share with low education | - 0.784^***^ | - 0.646^***^ | - 0.921^***^ | - 0.745^***^ | - 0.458^***^ | - 0.408^***^ | - 0.511^***^ | - 0.409^***^ |
|  | (0.111) | (0.085) | (0.096) | (0.084) | (0.096) | (0.095) | (0.098) | (0.095) |
|  |  |  |  |  |  |  |  |  |
| Constant | - 0.596 | 65.778^*^ | 0.781 | 31.177 | 57.067^*^ | 41.284 | 68.023^*^ | 43.059 |
|  | (32.269) | (28.264) | (32.616) | (28.270) | (27.090) | (26.970) | (28.055) | (26.900) |
| Observations | 290 | 290 | 290 | 290 | 290 | 290 | 290 | 290 |
| R^2^ | 0.726 | 0.800 | 0.720 | 0.792 | 0.820 | 0.826 | 0.807 | 0.828 |
| ***Notes:*** Unstandardized coefficients; robust standard errors within parentheses.  Significance: *p < 0.05; **p < 0.01; ***p < 0.001. All models include county-fixed effects. | | | | | | | | |
