# Supplementary material for: Variations in vaccination uptake: COVID-19 vaccination rates in Swedish municipalities
Source: PLOS Glob Public Health. 2022 Oct 20;2(10):e0001204. doi: 10.1371/journal.pgph.0001204 (PMC10022166; doi:10.1371/journal.pgph.0001204)
Supplement: S6 Table — (DOCX) [file pgph.0001204.s011.docx]

**S6 Table.** OLS-regression models without potentially confounding variables.

|  | **Model 1** | **Model 2** | **Model 3** | **Model 4** | **Model 5** | **Model 6** | **Model 7** | **Model 8** |
| --- | --- | --- | --- | --- | --- | --- | --- | --- |
| SD voter share | - 0.516^***^ |  |  |  | - 0.325^***^ | - 0.318^***^ | - 0.291^**^ | - 0.324^***^ |
|  | (0.061) |  |  |  | (0.055) | (0.053) | (0.056) | (0.054) |
| Election turnout |  | 0.992^***^ |  |  | 0.441^***^ | 0.561^***^ | 0.811^***^ | 0.484^***^ |
|  |  | (0.074) |  |  | (0.124) | (0.094) | (0.108) | (0.123) |
| Members in free church |  |  | -0.070 |  | - 0.160 | - 0.133 | - 0.177 | - 0.136 |
|  |  |  | (0.129) |  | (0.093) | (0.092) | (0.096) | (0.092) |
| Share Foreign-born |  |  |  | - 0.412^***^ | - 0.209^***^ |  |  |  |
|  |  |  |  | (0.034) | (0.052) |  |  |  |
| Share born outside Europe |  |  |  |  |  | - 0.310^***^ |  | - 0.317^***^ |
|  |  |  |  |  |  | (0.065) |  | (0.065) |
| Share born in Europe |  |  |  |  |  |  | - 0.026 | - 0.069 |
|  |  |  |  |  |  |  | (0.075) | (0.073) |
| **Control variables** |  |  |  |  |  |  |  |  |
| Unemployment rate | - | - | - | - | - | - | - | - |
|  |  |  |  |  |  |  |  |  |
| Log(median income) | - | - | - | - | - | - | - | - |
|  |  |  |  |  |  |  |  |  |
|  |  |  |  |  |  |  |  |  |
| Log(population size) | -0.305 | 0.809^***^ | 1.057^***^ | 1.612^***^ | 0.366 | 0.500^*^ | 0.082 | 0.511^*^ |
|  | (0.277) | (0.198) | (0.254) | (0.210) | (0.238 | (0.241) | (0.234) | (0.241) |
| Share with low education | - | - | - | - | - | - | - | - |
|  |  |  |  |  |  |  |  |  |
|  |  |  |  |  |  |  |  |  |
| Constant | 82.296^***^ | -25.716^***^ | 58.430^***^ | 61.987^***^ | 37.468^**^ | 24.443^**^ | 3.016 | 31.963^**^ |
|  | (3.712) | (6.648) | (2.866) | (2.329) | (12.130) | (9.256) | (11.053) | (12.157) |
| Observations | 290 | 290 | 290 | 290 | 290 | 290 | 290 | 290 |
| R^2^ | 0.560 | 0.666 | 0.485 | 0.664 | 0.738 | 0.744 | 0.722 | 0.745 |
| ***Notes:*** Unstandardized coefficients; robust standard errors within parentheses.  Significance: *p < 0.05; **p < 0.01; ***p < 0.001. All models include county-fixed effects. | | | | | | | | |
