# Supplementary material for: Variations in vaccination uptake: COVID-19 vaccination rates in Swedish municipalities
Source: PLOS Glob Public Health. 2022 Oct 20;2(10):e0001204. doi: 10.1371/journal.pgph.0001204 (PMC10022166; doi:10.1371/journal.pgph.0001204)
Supplement: S7 Table — (DOCX) [file pgph.0001204.s012.docx]

**S7 Table.** Beta-regression models.

|  | **Model 1** | **Model 2** | **Model 3** | **Model 4** | **Model 5** | **Model 6** | **Model 7** | **Model 8** |
| --- | --- | --- | --- | --- | --- | --- | --- | --- |
| SD voter share | -0.007^*^ |  |  |  | -0.010^**^ | -0.011^***^ | -0.008^*^ | -0.011^***^ |
|  | (0.003) |  |  |  | (0.003) | (0.003) | (0.003) | (0.003) |
| Election turnout |  | 0.027^***^ |  |  | 0.011 | 0.019^***^ | 0.022^***^ | 0.013 |
|  |  | (0.005) |  |  | (0.007) | (0.005) | (0.007) | (0.007) |
| Members in free church |  |  | -0.000 |  | -0.000 | -0.000 | -0.000 | -0.000 |
|  |  |  | (0.005) |  | (0.000) | (0.000) | (0.000) | (0.000) |
| Share Foreign-born |  |  |  | -0.011^***^ | -0.009^**^ |  |  |  |
|  |  |  |  | (0.002) | (0.003) |  |  |  |
| Share born outside Europe |  |  |  |  |  | -0.015^***^ |  | -0.015^***^ |
|  |  |  |  |  |  | (0.004) |  | (0.004) |
| Share born in Europe |  |  |  |  |  |  | -0.004 | -0.005 |
|  |  |  |  |  |  |  | (0.003) | (0.003) |
| **Control variables** |  |  |  |  |  |  |  |  |
| Unemployment rate | -0.011 | -0.008 | -0.010 | 0.003 | 0.001 | 0.010 | -0.009 | 0.009 |
|  | (0.006) | (0.006) | (0.006) | (0.006) | (0.007) | (0.008) | (0.006) | (0.008) |
| Log(median income) | 395^*^ | -0.017 | 0.381^*^ | 0.279 | 0.148 | 0.141 | 0.019 | 0.180 |
|  | (0.156) | (0.168) | (0.158) | (0.151) | (0.171) | (0.168) | (0.169) | (0.170 |
|  |  |  |  |  |  |  |  |  |
| Log(population size) | -0.034^**^ | -0.002 | -0.024^*^ | 0.003 | -0.005 | 0.002 | -0.012 | 0.002 |
|  | (0.012) | (0.012) | (0.012) | (0.012) | (0.012) | (0.013) | (0.012) | (0.013) |
| Share with low education | -0.040^***^ | -0.038^***^ | -0.048^***^ | -0.041^***^ | -0.027^***^ | -0.025^***^ | -0.030^***^ | -0.025^***^ |
|  | (0.007) | (0.006) | (0.006) | (0.006) | (0.007) | (0.007) | (0.007) | (0.007) |
|  |  |  |  |  |  |  |  |  |
| Constant | 3.187 | -0.767 | -3.138 | -2.029 | -1.276 | -2.028 | -0.659 | -1.937 |
|  | (2.010) | (1.985) | (2.030) | (1.937) | (1.939) | (1.955) | (1.964) | (1.948) |
| Observations | 290 | 290 | 290 | 290 | 290 | 290 | 290 | 290 |
| R^2^ | 0.735 | 0.753 | 0.730 | 0.754 | 0.766 | 0.768 | 0.759 | 0.769 |
